# SCP2-mediated cholesterol membrane trafficking promotes the growth of pituitary adenomas via Hedgehog signaling activation

**DOI:** 10.1186/s13046-019-1411-9

**Published:** 2019-09-13

**Authors:** Xiao Ding, Kexia Fan, Jintao Hu, Zhenle Zang, Shunli Zhang, Yin Zhang, Zhichao Lin, Xiangdong Pei, Xin Zheng, Feng Zhu, Hui Yang, Song Li

**Affiliations:** 1 Multidisciplinary Center for Pituitary Adenomas of Chongqing, Department of Neurosurgery, Xinqiao Hospital, Army Medical University, 183 Xinqiao Main Street, Shapingba District, Chongqing, 400038 China; 2 Lab of Innovative Drug Research and Bioinformatics, College of Pharmaceutical Sciences, Zhejiang University, Hangzhou, 310058 Zhejiang China

**Keywords:** Cholesterol, Growth, Hedgehog signaling, Pituitary adenomas, SCP2

## Abstract

**Background:**

Metabolic reprogramming is an important characteristic of tumors. In the progression of pituitary adenomas (PA), abnormal glucose metabolism has been confirmed by us before. However, whether cholesterol metabolism is involved in the process of PA remains unclear. This study aimed to investigate whether abnormal cholesterol metabolism could affect the progression of PA.

**Methods:**

We analyzed the expression of sterol carrier protein 2 (SCP2) in 40 surgical PA samples. In vitro experiments and xenograft models were used to assess the effects of SCP2 and cholesterol on proliferation of PA. The incidence of hypercholesterolemia between 140 PA patients and 100 heathy controls were compared.

**Results:**

We found an upregulation of SCP2 in PA samples, especially in tumors with high proliferation index. Forced expression of SCP2 promoted PA cell lines proliferation in vitro. Furthermore, SCP2 regulated cholesterol trafficking from cytoplasm to membrane in GH3 cells, and extracellularly treating GH3 cells and primary PA cells with methyl-β-cyclodextrin/cholesterol complex to mimic membrane cholesterol concentration enhanced cell proliferation, which suggested a proliferative effect of cholesterol. Mechanistically, cholesterol induced activation of PKA/SUFU/GLI1 signaling via smoothened receptor, which was well-known as Hedgehog signaling, resulting in inhibiting apoptosis and promoting cell cycle. Accordingly, activation of Hedgehog signaling was also confirmed in primary PA cells and surgical PA samples. In vivo, SCP2 overexpression and high cholesterol diet could promote tumor growth. Intriguingly, the incidence of hypercholesterolemia was significantly higher in PA patients than healthy controls.

**Conclusions:**

Our data indicated that dysregulated cholesterol metabolism could promote PA growth by activating Hedgehog signaling, supporting a potential tumorigenic role of cholesterol metabolism in PA progression.

## Background

Pituitary adenomas (PA) account for 10-15% of primary intracranial tumors [1]. In the clinic, some cases of PA characterized by rapid local growth and tumor extension show malignant features and poor prognosis [2]. However, specific definitions and classifications of this kind of PA according to the 2017 WHO classification of PA have not been conducted [3]. Because of their malignant features, these tumors often wrap around nerves and blood vessels, rendering total resection difficult. In addition, the postoperative pathology of these tumors usually shows an increased proliferation index (as evidenced by the expression of Ki-67 and P53, markers of tumor proliferative activity) [3]. Together, these characteristics result in early recurrence and secondary surgery, which confer a poor prognosis in patients. Understanding the factors affecting the occurrence and progression of these “high proliferation index PA” (HI-PA) is critical to developing new therapeutic methods and improving the prognosis of patients.

Reprogramming of cellular metabolism is an important characteristic of tumors [4]. We initially confirmed that lactate dehydrogenase A -mediated abnormal glucose metabolism promoted the proliferation and invasion of PA cells [5]. However, whether cholesterol metabolism is involved in the process of PA remains unclear. Increasing evidence supports the hypothesis that cholesterol plays an important role in tumor progression. For example, clinical research has reported that hypercholesterolemia is positively correlated with the risk of developing some types of cancer, such as breast, colorectal and prostate cancer [6–8]. A recent study showed that lysosomal cholesterol could activate mTORC1 via an SLC38A9-Niemann-Pick C1 signaling complex, indicating that cholesterol could directly activate oncogenic signaling [9]. Lipid rafts are a platform for signal transduction, and their structure and function are dependent on their cholesterol composition [10]. Disrupting the functions of lipid rafts by targeting the membrane cholesterol content has been shown to affect tumor progression and invasion [11]. Cholesterol is synthesized via the mevalonate pathway and regulated by HMG-CoA reductase (HMGCR), a key flux-controlling enzyme. The expression of HMGCR in tumor cells was found to be higher than that in untransformed cells, suggesting that dysregulation of the mevalonate pathway may have carcinogenic abilities that can drive malignant transformation and maintain tumor growth [12, 13]. Additionally, statins, as HMGCR inhibitors, may be beneficial for tumor prevention and therapy [14]. Moreover, tumor cells were shown to present with abnormal distributions of cellular cholesterol [15], and some key cholesterol transporters have been characterized as cancer-related factors. For example, sterol carrier protein 2 (SCP2) is well recognized as an intracellular cholesterol trafficking protein targeting cholesterol to cholesterol-rich membrane microstructural domains [16–18]. SCP2 expression has been reported to be related to the progression of gliomas, and downregulating SCP2 protein expression could suppress tumor cell proliferation by inducing autophagy [19]. However, whether SCP2 can affect the progression of PA by directly regulating cholesterol metabolism remains unknown.

Altogether, although abnormal cholesterol metabolism plays an important role in tumor progression, studies on the precise mechanisms are limited. In the current study, we investigated whether abnormal cholesterol metabolism affects the progression of PA. Our study offers new insight into the mechanism underlying PA progression and provides molecular mechanistic arguments for targeting cholesterol metabolism in PA treatment.

## Methods

### Patients

Forty human PA samples were collected from patients who underwent transsphenoidal surgery at the multidisciplinary center for PA in Chongqing, Xinqiao Hospital. The diagnosis of individual tumors was based on clinical and endocrine evaluation, with additional information gained from pathological evaluation. In the pathological evaluation, HI-PA tumors were identified by Ki-67 index ≥3%, accompanied by P53 positivity; low proliferation index PA (LI-PA) tumors were identified by Ki-67 index < 3%, accompanied by P53 negativity, according to the prognostic clinicopathological classification of PA described by Trouillas [20].

A total of 100 sets of plasma cholesterol data were collected from patients with PA who underwent transsphenoidal surgery at the multidisciplinary center for PA in Chongqing, Xinqiao Hospital. The inclusion criteria for the 100 PA patients were the absence of both pituitary dysfunction and a medication history of cholesterol-lowering drugs. A total of 140 sets of plasma cholesterol data were collected from healthy controls who underwent a routine physical examination at the physical examination center of Xinqiao Hospital. The inclusion criteria for the 140 healthy controls were the absence of PA on a CT scan and the absence of a medication history of cholesterol-lowering drugs.

Clinical data regarding the sex, age and PA hormone type of all patients are summarized in Additional file 1: Table S1-S3.

### Reagents

The following reagents were obtained: human pituitary gland RNA standard (Clontech, 636157), methyl-β-cyclodextrin (Mβ-CD; Sigma, C4555), methyl-β-cyclodextrin cholesterol complex (Mβ-CD/CHO; Sigma, C4951), itraconazole (MedChemExpress, HY-17514), vismodegib (MedChemExpress, HY-10440), forskolin (MedChemExpress, HY-15371), ezetimibe (MedChemExpress, HY-17376), Rapamycin (Beyotime, Nanjing, China, S1842).

Antibodies against the following proteins were used in this study: SCP2 (Abcam, ab140126), protein kinase A (PKA; Abcam, ab75991), suppressor of fused (SUFU; Proteintech, Wuhan, China, 26759-1-AP), glioma-associated oncogene 1 (GLI1; Santa Cruz, sc-515751 and sc-515781), B cell lymphoma 2 (BCL2; Santa Cruz, sc-7382), cyclin D1 (CCND1; Abcam, ab134175), phosphorylation-protein kinase B (p-AKT; Cell signaling technology, 4060), protein kinase B (AKT; Cell signaling technology, 4691), phosphorylation-Mammalian Target of Rapamycin (p-mTOR; Abcam, ab84400), Mammalian Target of Rapamycin (mTOR; Abcam, ab 32028), β-actin (Abcam, ab8226) and GH (growth hormone; Santa Cruz, sc-374266).

### Cell culture

Rat GH3 (ATCC, CCL-82.1) and MMQ (ATCC, CRL-10609) and mouse AtT-20 (ATCC, CCL-89) PA cells were cultured in Ham’s F-12 K medium (GIBCO, 21127-022) containing 2.5% fetal bovine serum (GIBCO, 10100-147) and 15% horse serum (GIBCO, 16050-122) in a humidified atmosphere of 5% CO2 at 37 °C.

To obtain primary human PA cells, human PA specimens were harvested from patients undergoing transsphenoidal surgery at Xinqiao Hospital in Chongqing, China (Additional file 1: Table S4). Fresh tissues were washed with PBS and minced into small pieces. Tissue fragments were then digested with type I collagenase for 2 hours at 37 °C. An equal volume of MEM (GIBCO, 11095-080) supplemented with 10% fetal bovine serum was added, and the cell suspension was filtered through a 200 Mo filter. After two washes with PBS, cells were resuspended in complete MEM and cultured in a humidified atmosphere of 5% CO2 at 37 °C.

### Lentiviral and shRNA transfection

**Fig. 1:**
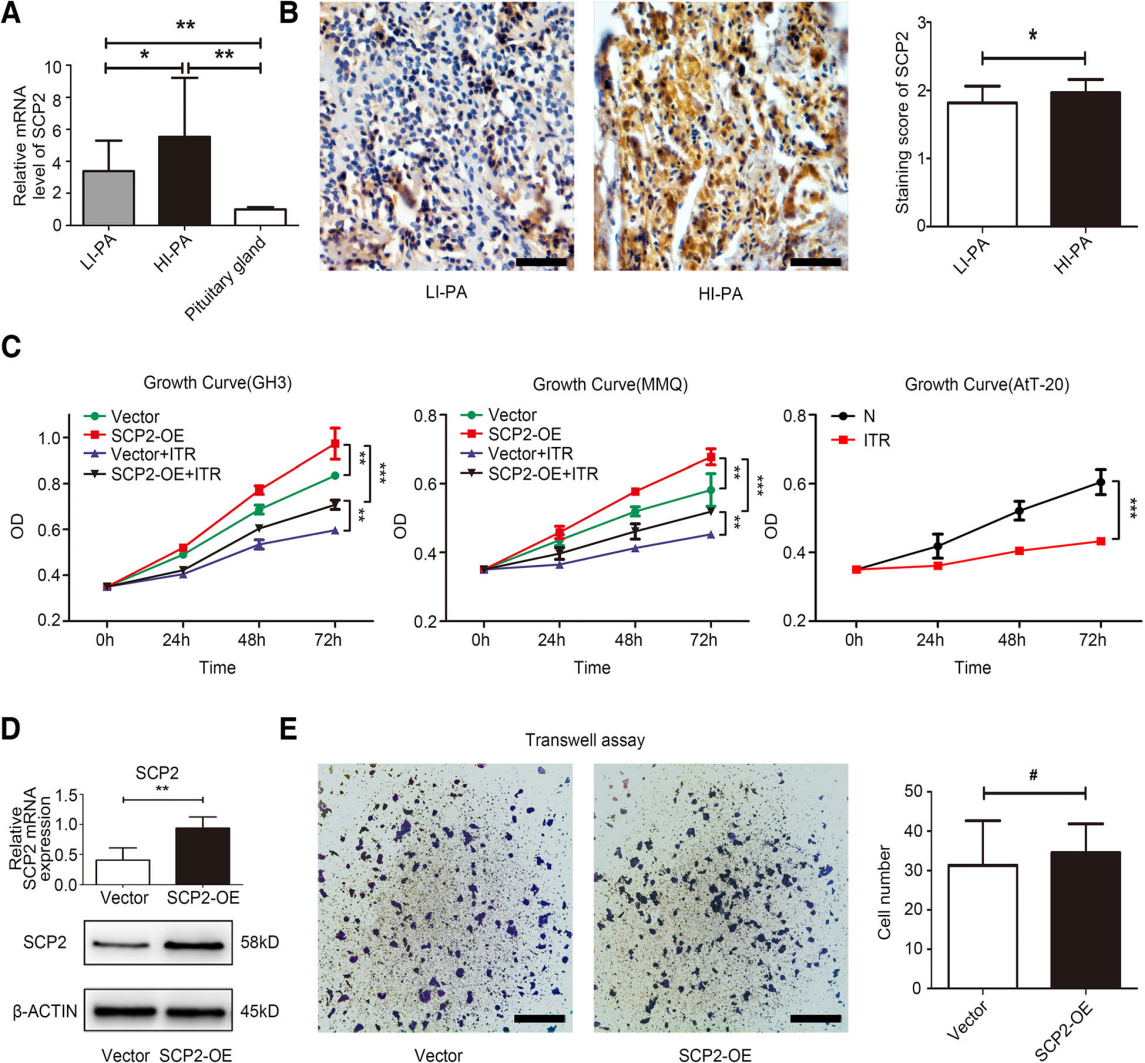
Expression of SCP2 in human PA samples and forced expression of SCP2 promoted the proliferation of PA cell lines. **a** Expression of SCP2 mRNA in low proliferation index PA (LI-PA) human tissues, high proliferation index PA (HI-PA) human tissues and human pituitary gland RNA standard was assessed by qRT-PCR. **b** Expression of the SCP2 protein in human PA samples was assessed by IHC staining (left panel). Statistical analysis of the IHC staining (right panel). Scale bar, 50 μm. **c** Control (Vector) and SCP2-overexpressing (SCP2-OE) GH3 and MMQ cells were incubated with 10 μM itraconazole (ITR) for 0, 24, 48 or 72 h, and cell proliferation of the four groups (Vector, SCP2-OE, Vector + ITR and SCP2-OE + ITR) at different time points was assessed by a CCK-8 assay (left two line charts, *n* = 6, ± SEM). Wild-type (N) AtT-20 cells was incubated with 10 μM itraconazole (ITR) for 0, 24, 48 or 72 h, and cell proliferation of the two groups (N and N + ITR) at different time points was assessed by a CCK-8 assay (right line chart, n = 6, ± SEM). **d** SCP2 mRNA and protein levels were measured in GH3 cells (Vector and SCP2-OE) by RT-qPCR and Western blotting (*n* = 3, ± SEM). **e** Invasion of Vector and SCP2-OE cells was evaluated by a Transwell assay. Scale bar, 100 μm (n = 3, ± SEM). An unpaired t-test was used to assess statistical significance. *P < 0.05; **P < 0.01; ***P < 0.001; #, not significant

Empty and SCP2 overexpression lentiviral vectors (ObiO, Shanghai, China) were transfected into cells at a multiplicity of infection of 100 according to the manufacturer’s instructions. Stable colonies were selected by adding puromycin (5 μg/ml) to the transfectants for 72 h and separating cells with fluorescent labeling by flow cytometry. Transfection efficiency was determined by real-time PCR and Western blotting (Fig. 1d).

Cells were transfected with negative control or smoothened (SMO) knockdown shRNA vectors (Genechem, Shanghai, China) using Lipofectamine 2000 (Invitrogen Life Technologies, 11,668-027) according to the manufacturer’s instructions. Fluorescence labeled cells were isolated by flow cytometry. Transfection efficiency was determined by real-time PCR (Additional file 2: Figure S2C).

### Cell proliferation assay

A WST-8 Cell Counting Kit-8 (CCK-8; Dojindo, CK04) was used to measure cell proliferation. Cells were seeded at 1 × 10^4^ cells/well into 96-well plates with 100 μl culture medium. The 10 μl of CCK-8 reagent was added to the wells at specific time points and incubated for 2 h at 37 °C. The reaction product was quantified according the manufacturer’s instructions.

### Invasion assay

Transwell chambers (Costar, 3422) were precoated with 100 μl of Matrigel (Corning, 356234) for 30 min at 37 °C. Approximately 5 × 10^4^ cells were starved in serum-free medium for 24 h and then trypsinized, resuspended in 200 μl of serum-free medium and placed into the upper chamber. Five hundred μl of F-12 K complete medium was added to the lower chamber. After incubation with the indicated treatments for 48 h, cells that invaded to the other side of the membrane were stained with crystal violet staining solution (Beyotime, C0121) and then imaged. Cell numbers in 5 different fields of view were counted under a light microscope at 200 × magnification.

### Immunohistochemistry

Tumor tissue was fixed in 4% paraformaldehyde for 24 h and embedded in paraffin. Embedded tumor tissues were sectioned at 5 μm for subsequent Immunohistochemical (IHC) staining. IHC staining was performed according to standard protocols. Anti-SCP2 (1:50), anti-PKA (1:100), anti-SUFU (1:50), and anti-GLI1 (Santa Cruz, sc-515781, 1:50) antibodies were used. Negative controls were performed completely according to the standard protocols we previously reported [21], including omission of the primary antibody by PBS and incubation with an isotype specific control IgG of rabbit (Abcam, ab172730) or mouse (Santa Cruz, sc-2025). No immunoreactive cells were detected in negative control experiments (Additional file 7: Figure S7).

The immunoreactivity of SCP2, PKA, SUFU and GLI1 was evaluated as previously reported [22, 23]. A Leica DM IRB microscope with a square grid inserted into the eyepiece was used to examine an area of 781.250 μm^2^ (200 high power nonoverlapping fields with widths of 0.0625 × 0.0625 mm). The staining intensity was assessed using a semiquantitative three point scale within the scope of the IR and was defined as absent (−, 0), weak (+, 1), moderate (++, 2), or strong (+++, 3). These scores represented the predominant staining intensity in each section and were calculated as an average of the selected samples.

### Filipin staining and immunofluorescence

Filipin III (Cayman, 70440) was dissolved in DMSO to a final concentration of 5 mg/ml. Cultured cells were fixed with 4% paraformaldehyde for 30 min at room temperature and stained with 50 μg/ml filipin III for 2 h at 4 °C in the dark.

Immunofluorescence was performed according to standard protocols. 4% paraformaldehyde fixed cells were incubated overnight with primary antibody against Anti-GH (1:50). After washing three times with PBS, cells were stained with FITC-conjugated secondary antibody (1:500, Beyotime, A0568). Antifade mounting medium with propidium iodide (PI; Beyotime, P0135) was used for nuclear staining and fluorescence decay resistance.

### Cholesterol determination

The cholesterol levels were quantified according to protocols from previous reports, with minor modifications [19, 24]. The total plasma cholesterol level of nude mice and the total cellular cholesterol level were quantified using a Total Cholesterol Assay Kit (Applygen, Beijing, China, E1015) according to the manufacturer’s protocol. To quantify intracellular cholesterol, cells were fixed with 0.1% glutaraldehyde for 30 min and treated with 2 U/ml cholesterol oxidase (Sigma, C5421) for 15 min to oxidize plasma membrane cholesterol. Then, methanol/chloroform (vol:vol, 1:2) was used to extract intracellular cholesterol, which was quantified using the Total Cholesterol Assay Kit. The plasma membrane cholesterol content was obtained by subtracting the intracellular cholesterol value from the total cellular cholesterol value. For relative quantification, the content of total cellular cholesterol in each group was normalized to that in the control group.

### Flow cytometry

For cell cycle analysis, cells were collected at the indicated time points for the relevant treatments and fixed in 70% ethanol at 4 °C overnight. Cells were resuspended and stained in 1 ml of PBS containing 50 μg/ml PI (BD Biosciences Pharmingen, 556463) and were then analyzed by flow cytometry (FACScould; BD Biosciences Pharmingen). For cell apoptosis analysis, cells were collected at the indicated time points for the relevant treatments. Apoptosis was assessed using a FITC-Annexin V Apoptosis Detection Kit (BD Biosciences Pharmingen, 556547) based on the manufacturer’s instructions, and cells were detected by flow cytometry.

### Reverse transcription and qPCR

Total RNA was extracted using TRIzol Reagent (TaKaRa, Dalian, China, 9108), and 1 μg of total RNA was reverse transcribed to cDNA using a PrimeScript RT Reagent Kit (TaKaRa, PR047A). TB Green Premix Ex Taq II (TaKaRa, RR820A) and a CFX96 Real-time System (Bio-Rad Laboratories) were used to carry out qPCR. The relative expression levels were calculated using the 2 − ^△△ct^ method. The primer sequences used for qPCR are listed in Additional file 1: Table S5.

### Western blotting analysis

Cell extracts containing 50 μg of protein were subjected to SDS-PAGE (Boster, Wuhan, China, AR0138) and transferred to PVDF membranes (Millipore, ISEQ00010). After blocking, membranes were incubated overnight with primary antibodies against SCP2 (1:1000), PKA (1:2500), SUFU (1:1000), GLI1 (Santa Cruz, sc-515751, 1:100), BCL2 (1:200), CCND1 (1:1000), p-AKT (1:1000), AKT (1:1000), p-mTOR (1:1000), mTOR (1:1000) and β-actin (1:5000). Membranes were probed with secondary antibodies (1:2000; Zsbio, Beijing, China, ZB-2305 and ZB-5301). The signals from the membrane were detected by enhanced chemiluminescence.

### In vivo experiments

For the in vivo xenograft experiments, 4-week-old male BALB/cA-nu mice were purchased from Beijing HFK Bioscience Co. Ltd. (Beijing, China) and housed under SPF conditions. A total of 4 × 10^6^ transfected cells suspended in 100 μl of solution (50% PBS and 50% Matrigel) were subcutaneously inoculated into the right flank of the mice. A high-cholesterol diet (HCD; standard chow containing 3% cholesterol and 0.5% cholic acid) was started after cell implantation [25]. Treatment with ezetimibe and vismodegib was started 9 days after cell implantation. Then, the tumor-bearing animals were divided into 5 groups (5 mice/group) according to the different treatments. Mice in the HCD-treated groups (Vector + HCD and Vector + HCD + ezetimibe) were fed high-cholesterol chow until sacrifice, while mice in the other groups were fed standard chow. Mice in the ezetimibe-treated group (Vector + HCD + ezetimibe) received a daily intraperitoneal injection of 30 mg/kg ezetimibe and mice in the vismodegib-treated group (SCP2-OE + vismodegib) received a daily intraperitoneal injection of 25 mg/kg vismodegib for the next 3 weeks until sacrifice [26, 27], while mice in the other groups received a daily intraperitoneal injection of an equal volume of PBS. The tumor volumes were measured every 3 days. Tumor volumes were calculated using the following formula: V (mm^3^) = [AB^2^]/2, where A is the tumor length and B is the tumor width. Tumor tissue was removed and blood was collected from the tumor-bearing mice following the final treatment. All animal procedures were conducted according to protocols approved by the Institutional Animal Care and Ethics Committee.

### Statistical analysis

The data are expressed as the means ± SEMs. The correlations between hypercholesterolemia and the occurrence of PA were determined by a chi-squared test. A two-tailed Student’s t-test was applied to determine statistical significance between the two groups. These analyses were performed using SPSS for Windows, version 13.0 (SPSS Inc., USA).

## Results

### SCP2 expression was upregulated in PA samples and SCP2 overexpression promoted the proliferation of PA cell lines

First, we investigated the expression of SCP2 in 40 surgical PA samples and compared it with that in the normal pituitary gland. The RT-qPCR results indicated that the expression of SCP2 mRNA was dramatically upregulated in PA samples compared with that in the normal pituitary gland (RNA standard). Moreover, the SCP2 mRNA level was higher in HI-PA samples than in LI-PA samples (Fig. 1a). Further IHC results indicated that the SCP2 protein was mainly localized in the cytoplasm of tumor cells and that HI-PA samples exhibited a higher staining intensity than LI-PA samples (Fig. 1b, left panel). The statistical results confirmed the upregulated expression of SCP2 in HI-PA samples (Fig. 1b, right panel), which suggested a proliferation-related role for SCP2 in PA cells. Consequently, in vitro cell experiments were performed to verify our hypothesis. The results of the CCK-8 assay confirmed that overexpression of SCP2 enhanced the proliferation of GH3 and MMQ rat PA cell lines. In addition, itraconazole, a specific inhibitor of SCP2 [19], effectively inhibited the protein expression of SCP2 (Additional file 2: Figure S2A) and significantly attenuated the proliferation of these two cell lines (Fig. 1c, left two line charts). Additionally, itraconazole significantly inhibited the proliferation of another cell line, AtT-20 mouse PA cells (Fig. 1c, right line chart). Enforced expression of SCP2 in GH3 cell lines by lentiviral vector delivery resulting in stable upregulation of SCP2 expression was confirmed by RT-qPCR and Western blotting (Fig. 1d). Tumor proliferation and invasion are closely related, thus, we further investigated whether SCP2 could affect PA cell invasion. The results of transwell assay showed that overexpression of SCP2 did not affect the invasion of GH3 cells (Fig. 1e).

### SCP2 promoted the proliferation of GH3 cells by regulating abnormal cholesterol membrane trafficking

**Fig. 2:**
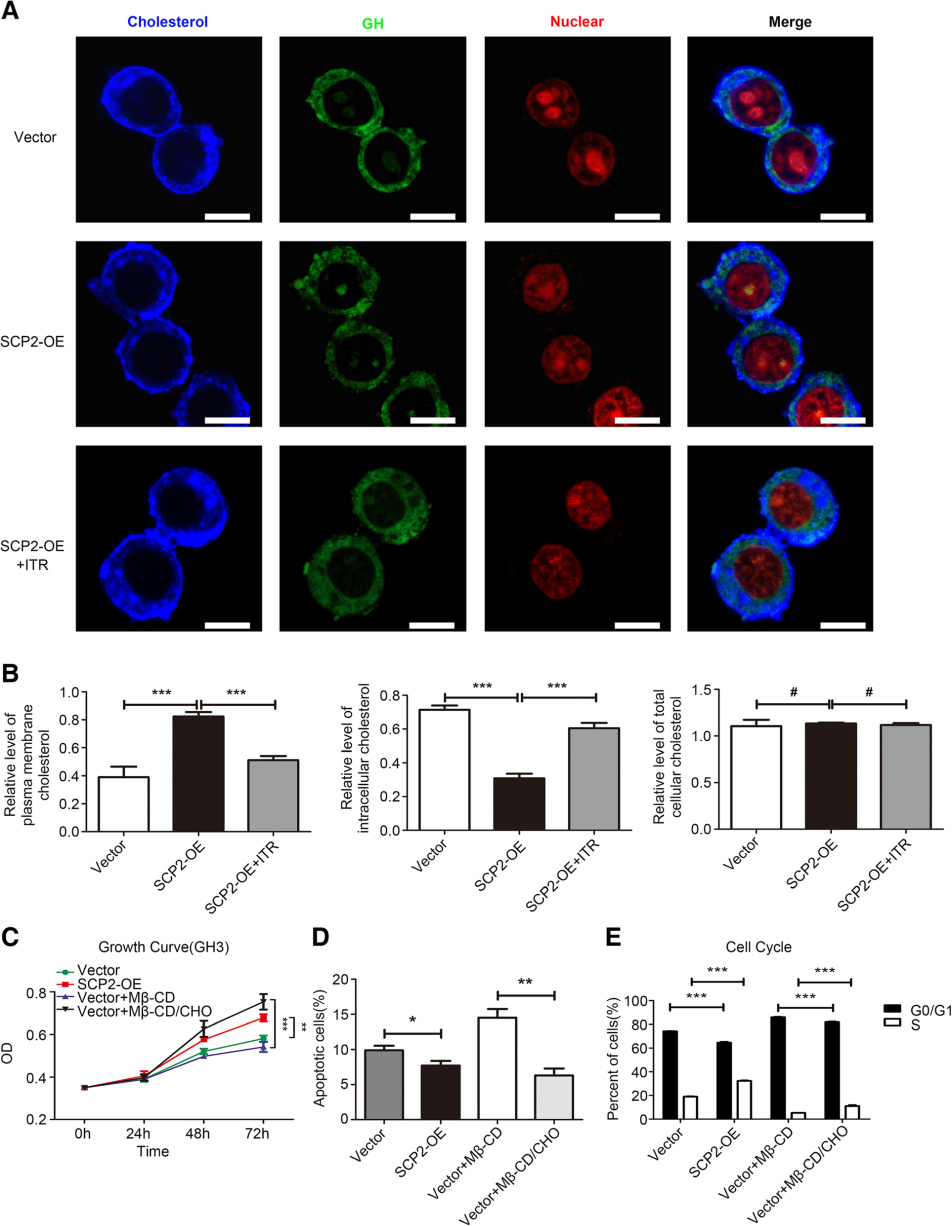
SCP2 regulated abnormal cholesterol membrane trafficking affected cell proliferation, apoptosis and cell cycle in GH3 cells. **a** SCP2-OE GH3 cells were incubated with 10 μM itraconazole (ITR) for 48 h. The distribution of cholesterol (blue) in the different groups (Vector, SCP2-OE and SCP2-OE + ITR) were examined by filipin staining and laser confocal microscopy. Green signal, GH staining; red signal, PI nuclear staining. Scale bar, 10 μm. **b** SCP2-OE GH3 cells were incubated with 10 μM itraconazole (ITR) for 48 h. Total cellular cholesterol content was measured using the Cholesterol Assay Kit. Plasma membrane and intracellular cholesterol levels were quantified using the cholesterol oxidation-based method (*n* = 12, ± SEM). **c** Vector GH3 cells were treated with Mβ-CD (20 μg/ml) or the Mβ-CD/CHO complex (20 μg/ml) for 0, 24, 48 or 72 h, and cell proliferation of the four groups (Vector, SCP2-OE, Vector + Mβ-CD and Vector + Mβ-CD/CHO) at different time points was assessed by a CCK-8 assay (n = 6, ± SEM). **d** Vector GH3 cells were treated with Mβ-CD (20 μg/ml) or the Mβ-CD/CHO complex (20 μg/ml) for 48 h. Cell apoptosis in the four groups (Vector, SCP2-OE, Vector + Mβ-CD and Vector + Mβ-CD/CHO) was measured by flow cytometry (n = 3, ± SEM). **e** The cell cycle distribution in the above four groups was analyzed using flow cytometry (n = 3, ± SEM). An unpaired t-test was used to assess statistical significance. *P < 0.05; **P < 0.01; ***P < 0.001; #, not significant.

SCP2 has been confirmed to play an important role in cholesterol trafficking, however, whether it plays a similar role in PA cells remains unknown. A fluorescent reagent, filipin III, which can bind specifically to unesterified cholesterol, was applied to label cellular cholesterol [19]. Considering the cell line could secret GH, GH was used as a cytoplasmic marker in staining experiment. We found that cholesterol was labeled with filipin III and distributed in the cytoplasm and plasma membrane of GH3 cells (Fig. 2a). Furthermore, the cholesterol content in whole cell, cytoplasmic and plasma membrane fractions was quantified as previously reported [24]. The levels of membrane cholesterol were significantly higher and the levels of intracellular cholesterol were much lower in SCP2-overexpressing GH3 cells than in control cells (Fig. 2b). Accordingly, inhibiting SCP2 by itraconazole significantly decreased the membrane cholesterol content and increased the intracellular cholesterol content relative to that in control cells. However, the total cholesterol content of cells was not affected by alterations in SCP2 levels. The above results confirmed that SCP2 could transport cholesterol from the cytoplasm to the plasma membrane without affecting the total cholesterol content of the cell.

We further investigated whether the effect of SCP2 on cell proliferation was due to abnormal cholesterol trafficking. Addition of the Mβ-CD/CHO complex has been shown to be the most effective approach to rapidly increase membrane cholesterol content [24, 28]. We treated GH3 cells with the Mβ-CD/CHO complex to mimic the membrane cholesterol concentration, an experiment designed as a positive control for SCP2 overexpression. The results of the CCK-8 assay showed that both overexpression of SCP2 (endogenous mode) and treatment with the Mβ-CD/CHO complex (exogenous mode) promoted the proliferation of GH3 cells (Fig. 2c). Furthermore, we used flow cytometry to explore whether the membrane cholesterol concentration affected cell apoptosis and the cell cycle. We found that both of the above modes significantly decreased the percentage of apoptotic cells and induced S phase transition (Fig. 2d, Additional file 4: Figure S4A, 2e and Additional file 5: Figure S5A). These results suggested that SCP2-mediated cholesterol membrane trafficking could promote cell proliferation by affecting apoptosis and the cell cycle.

### Abnormal cholesterol trafficking promoted the proliferation of GH3 cells by activating the Hedgehog signaling pathway

**Fig. 3:**
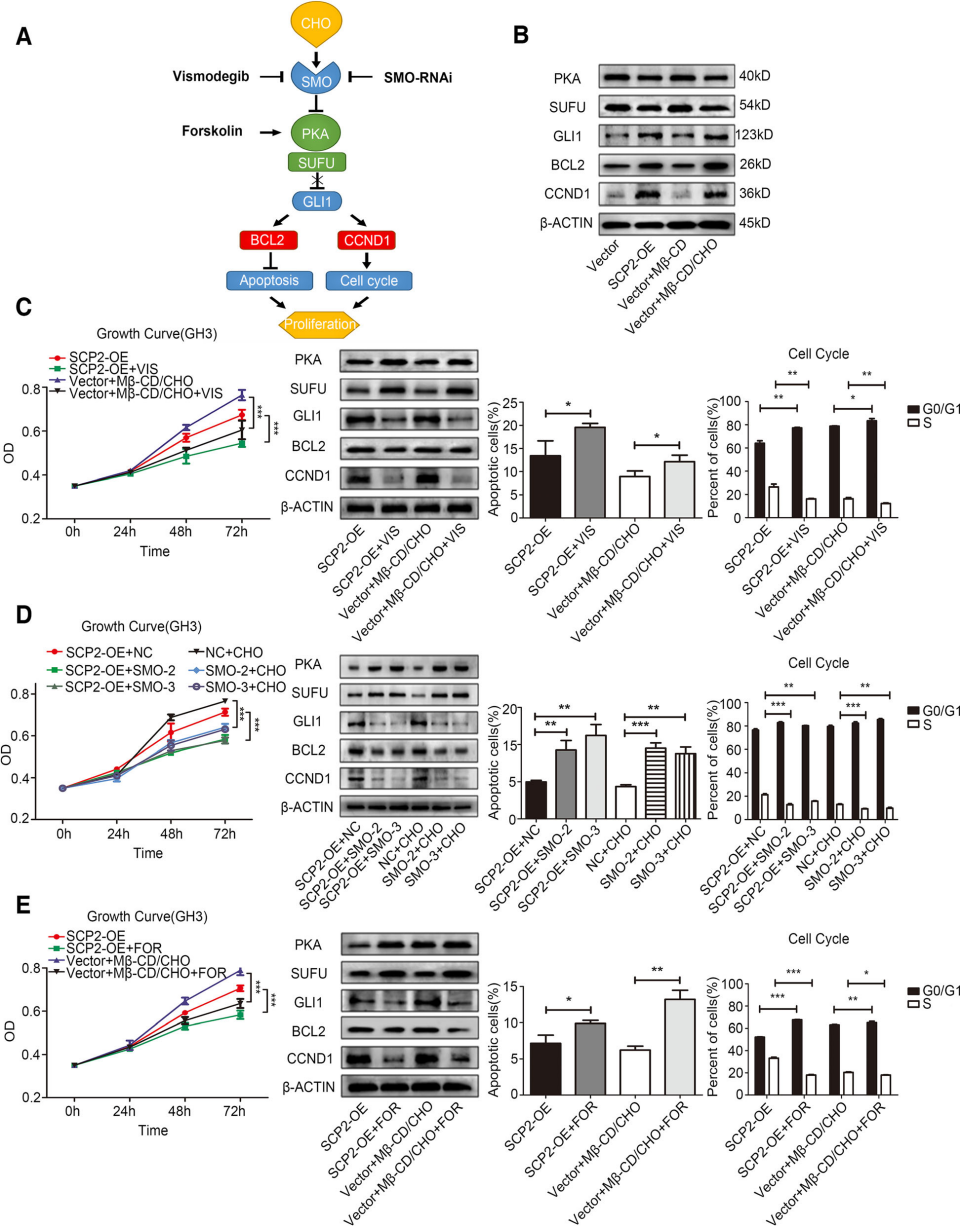
Membrane cholesterol promoted the proliferation of GH3 cells by activating the Hh signaling pathway. **a** A schematic showing the process via which cholesterol promotes cell proliferation by activating the Hh signaling pathway. The Mβ-CD/CHO complex (CHO) is an agonist and vismodegib is an antagonist that binds and modulates the activity of SMO. SMO RNAi was used to knock down SMO expression. Forskolin blocked signaling by elevating cAMP levels, which increased PKA activity. **b** Vector GH3 cells were treated with Mβ-CD (20 μg/ml) or the Mβ-CD/CHO complex (20 μg/ml) for 48 h. Protein expression levels of PKA, SUFU, GLI1, BCL2 and CCND1 in the four groups (Vector, SCP2-OE, Vector + Mβ-CD and Vector + Mβ-CD/CHO) were assessed by Western blotting. **c** SCP2-OE GH3 cells were treated with vismodegib (VIS, 50 μM) and Vector GH3 cells were treated with the Mβ-CD/CHO complex (20 μg/ml) in the presence or absence of vismodegib (50 μM) for 0, 24, 48 or 72 h. Cell proliferation in the four groups (SCP2-OE, SCP2-OE + VIS, Vector + Mβ-CD/CHO and Vector + Mβ-CD/CHO + VIS) at different time points was assessed by a CCK-8 assay (n = 6, ± SEM). Protein expression levels of PKA, SUFU, GLI1, BCL2 and CCND1 at 48 h were assessed by Western blotting. Cell apoptosis and the cell cycle at 48 h were analyzed using flow cytometry (n = 3, ± SEM). **d** SCP2-OE and wild-type GH3 cells were infected with negative control vector (NC) or SMO shRNA vector (SMO-2 and SMO-3) for 48 h, and the transfected wild-type GH3 cells were incubated with the Mβ-CD/CHO complex (CHO, 20 μg/ml) for another 0, 24, 48 or 72 h. Cell proliferation in the six groups (SCP2-OE + NC, SCP2-OE + SMO-2, SCP2-OE + SMO-3, NC+ CHO, SMO-2 + CHO and SMO-3 + CHO) at different time points were assessed by a CCK-8 assay (n = 6, ± SEM). Protein expression levels of PKA, SUFU, GLI1, BCL2 and CCND1 at 48 h were examined by Western blotting. Cell apoptosis and the cell cycle at 48 h were analyzed using flow cytometry (n = 3, ± SEM). **e** SCP2-OE GH3 cells were treated with forskolin (FOR, 25 μM) and Vector GH3 cells were treated with the Mβ-CD/CHO complex (20 μg/ml) in the presence or absence of forskolin (25 μM) for 0, 24, 48 or 72 h. Cell proliferation in the four groups (SCP2-OE, SCP2-OE + FOR, Vector + Mβ-CD/CHO and Vector + Mβ-CD/CHO + FOR) at different time points was assessed by a CCK-8 assay (n = 6, ± SEM). Protein expression levels of PKA, SUFU, GLI1, BCL2 and CCND1 at 48 h were assessed by Western blotting. Cell apoptosis and the cell cycle at 48 h were analyzed using flow cytometry (n = 3, ± SEM). An unpaired t-test was used to assess statistical significance. *P < 0.05; **P < 0.01; ***P < 0.001

Studies have reported that membrane cholesterol can directly activate the Hedgehog (Hh) signaling pathway by binding to a membrane receptor called SMO [29, 30]. Hh signaling is closely related to organ development, cell differentiation, cell proliferation, post-injury regeneration and tumor formation [31]. We hypothesized that the membrane cholesterol concentration-induced proliferation of GH3 cells might be due to the activation of Hh signaling. A simple schematic of the Hh signaling pathway and modulated the activation of the signaling pathway at different levels by different approaches were designed and presented (Fig. 3a). First, the western blotting results indicated that both the endogenous and exogenous modes could activate the Hh signaling pathway by decreasing the expression of PKA and SUFU, leading to increased expression of GLI1. Increased expression of GLI1 directly influenced the expression of two important downstream molecules, BCL2 and CCND1, which are related to apoptosis and the cell cycle (Fig. 3b, Additional file 3: Figure S3A). These results further explained the specific mechanism underlying the changes in apoptosis and the cell cycle described above.

Vismodegib, a specific inhibitor of the Hh signaling pathway, can specifically bind to SMO and inhibit its function to block the signaling pathway at the level of SMO [32] (Additional file 2: Figure S2B). The CCK-8 assay and Western blotting results showed that after treatment with vismodegib, the effects of both the endogenous and exogenous modes of promoting cell proliferation and Hh signaling pathway activation were reversed. The altered expression of BCL2 and CCND1 and the associated flow cytometry results further explained the suppressed cell proliferation (Fig. 3c, Additional file 3: Figure S3B, Additional file 4: Figure S4B and Additional file 5: Figure S5B). These results proved the key role of SMO in the Hh signaling pathway at the level of protein function.

To further confirm the effect of SMO, shRNA was used to knock down the expression of SMO, and stable downregulation of SMO was confirmed by RT-qPCR (Additional file 2: Figure S2C). The CCK-8 assay and western blotting results showed that downregulation of SMO expression reversed the effects of the endogenous and exogenous modes of promoting cell proliferation and activation of the Hh signaling pathway, which verified the results at the level of protein expression. Moreover, SMO shRNA affected apoptosis and the cell cycle by decreasing the expression of BCL2 and CCND1 (Fig. 3d, Additional file 3: Figure S3C, Additional file 4: Figure S4C and Additional file 5: Figure S5C).

Finally, forskolin was used to study the function of PKA in the signaling pathway. Forskolin, a cyclic adenosine monophosphate (cAMP) agonist, induced increased expression of PKA (Additional file 2: Figure S2D), blocking the Hh signaling pathway between SMO and GLI1 [29]. The CCK-8 assay and western blotting results indicated that upregulation of PKA expression reversed the effects of the endogenous and exogenous modes on the promotion of cell proliferation and Hh signaling pathway activation. These results confirmed that PKA is the key molecule in the signaling pathway, the change in PKA expression directly affected signaling pathway activation, apoptosis and the cell cycle (Fig. 3e, Additional file 3: Figure S3D, Additional file 4: Figure S4D and Additional file 5: Figure S5D).

Taken together, the above results suggested that abnormally trafficked cholesterol mediated by SCP2 could bind to the SMO receptor and activate the Hh signaling pathway, leading to upregulated expression of the apoptosis-related molecule BCL2 and the cell cycle-related molecule CCND1, thus promoting the proliferation of PA cells.

### Cholesterol promoted the proliferation of human primary PA cells by activating the Hh signaling pathway

**Fig. 4:**
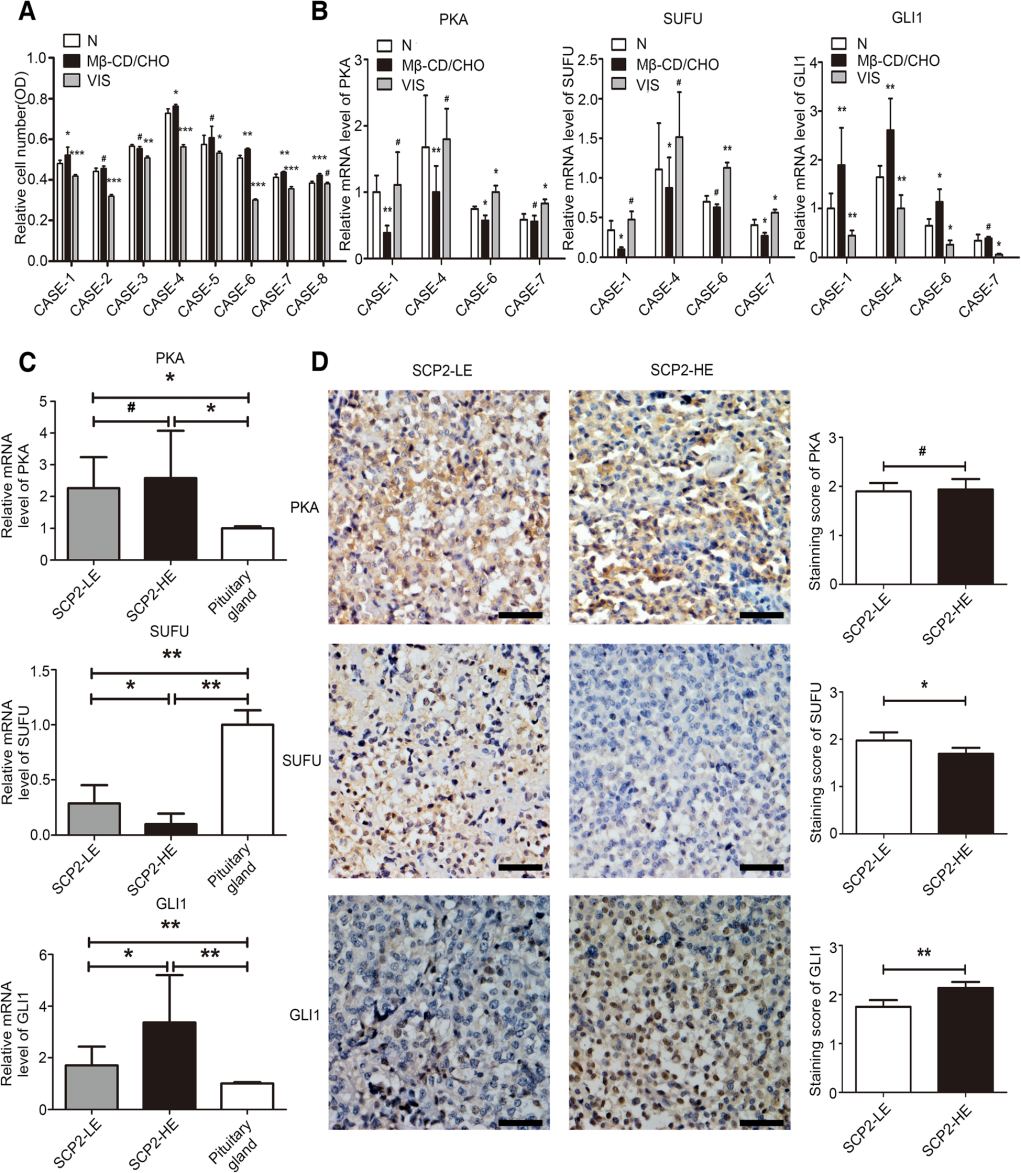
Activation of Hh signaling pathway in primary cultured human PA cells and human PA samples. **a** Primary cultured human PA cells were treated with the Mβ-CD/CHO complex (20 μg/ml) or vismodegib (VIS, 50 μM) for 48 h. Cell proliferation in three groups (N, Mβ-CD/CHO and VIS) from different cases was assessed by a CCK-8 assay. **b** The mRNA expression levels of PKA, SUFU and GLI1 were assessed by qRT-PCR at 48 h in the above three groups from Case 1, 4, 6 and 7. **c** The expression of PKA, SUFU and GLI1 mRNA in human PA samples with low and high expression of SCP2 (SCP2-LE and SCP2-HE, respectively) and human pituitary gland RNA standard was assessed by qRT-PCR. **d** PKA, SUFU and GLI1 protein expression in human PA samples with SCP2-LE and SCP2-HE was assessed by IHC staining (left panels). Statistical analysis of the IHC staining (right panels). Scale bar, 50 μm. An unpaired t-test was used to assess statistical significance. *P < 0.05; **P < 0.01; ***P < 0.001; #, not significant.

To further investigate the effect of cholesterol and the Hh signaling pathway on the proliferation of primary PA cells, we cultured primary cells derived from human GH-producing PA after transsphenoidal resection (Additional file 2: Figure S2E). Similar to the CCK-8 assay results with PA cell lines, the Mβ-CD/CHO complex promoted cell proliferation, while the signaling inhibitor vismodegib suppressed cell proliferation (Fig. 4a). The sensitive samples were selected for assessment of the mRNA level of Hh signaling molecules after treatment. The RT-qPCR results showed that after treatment with the Mβ-CD/CHO complex, the mRNA levels of PKA and SUFU decreased, while that of GLI1 increased. In addition, the mRNA level of GLI1 significantly decreased after vismodegib treatment, with an increase in PKA and SUFU mRNA levels (Fig. 4b). These results showed that cholesterol promoted the proliferation of human PA cells by activating the Hh signaling pathway, which were consistent with the results in the GH3 cell line.

### Activation of Hh signaling pathway was detected in human surgical PA samples

Since cholesterol has been demonstrated to activate the Hh signaling pathway in PA cell lines and primary human PA cells, we considered that signaling in human PA samples could also be activated. After reordering the PA samples according to SCP2 mRNA and protein expression, we selected samples with high expression (SCP2-HE) and low expression (SCP2-LE) for further investigation. RT-qPCR studies on signaling molecules indicated that the expression level of GLI1 was higher but the expression level of SUFU was lower in SCP2-HE PA samples than in SCP2-LE samples, with no change in PKA expression (Fig. 4c). In addition, the expression levels of PKA and GLI1 were higher but the expression level of SUFU was lower in PA samples than in the normal pituitary gland (Fig. 4c). The results of further IHC studies of these three molecules indicated that the PKA and SUFU protein were mainly localized in the cytoplasm of tumor cells, while the GLI1 protein was mainly localized in the cytoplasm and nucleus (Fig. 4d, left panels). The statistical results indicated that SUFU expression was up-regulated in SCP2-LE samples, GLI1 expression was up-regulated in SCP2-HE samples, and there was no statistical difference in PKA expression (Fig. 4d, right panels). These findings in PA samples further confirmed the previous experimental results.

### SCP2 and hypercholesterolemia regulated GH3 cell growth in vivo

**Fig. 5:**
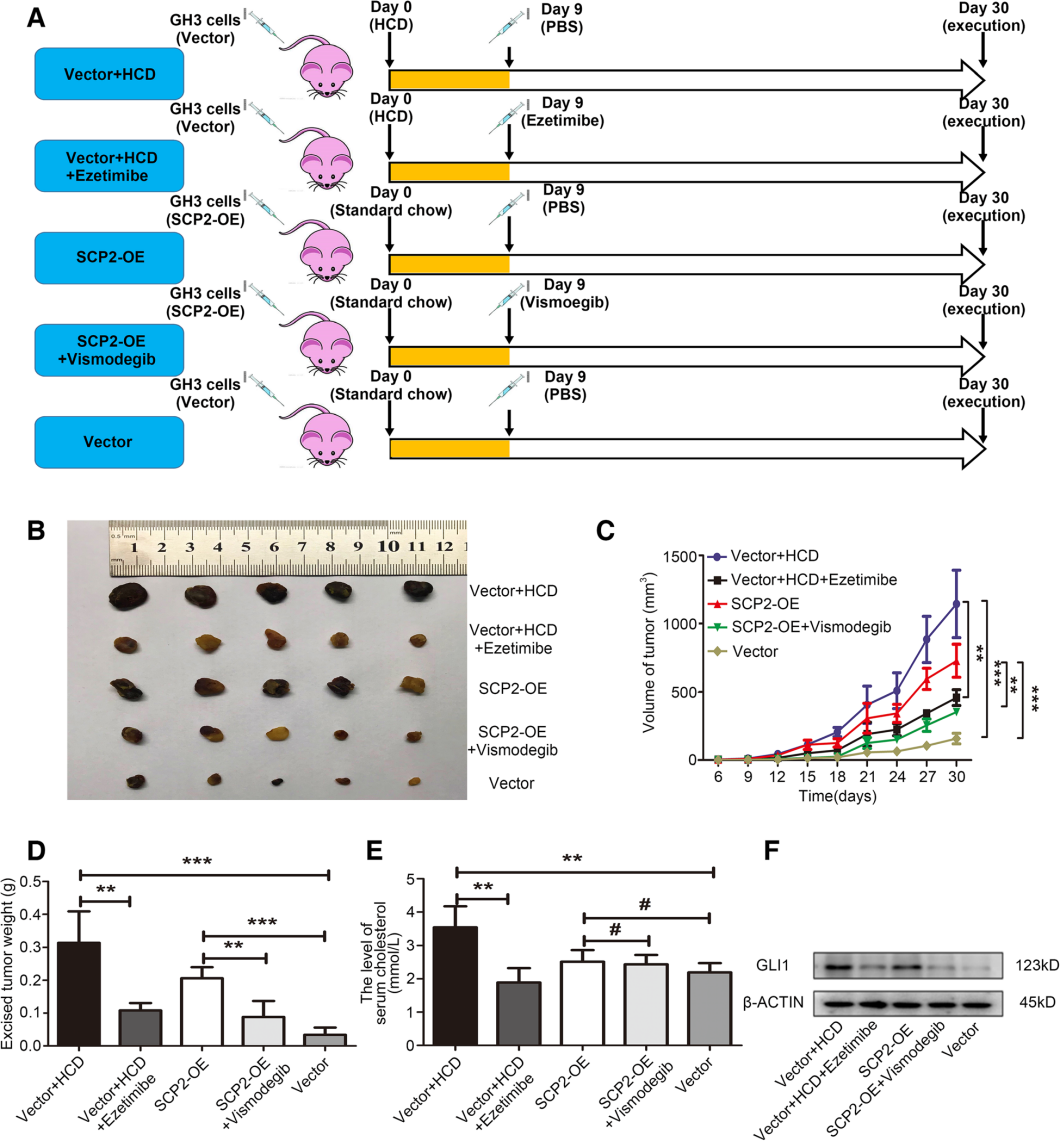
SCP2 and hypercholesterolemia regulated PA cell growth in vivo. **a** Schematic of dietary challenge and medication administration in mice injected with either Vector or SCP2-OE GH3 cells. HCD stands for high cholesterol diet. **b** Excised tumors in different groups are shown. **c** Growth curve showing the changes in the tumor volume in mice after different treatments. **d** Weight of the excised tumors in each group. **e** Plasma cholesterol level in mice in each group after sacrifice. **f** Protein expression level of GLI1 in excised tumors were examined by Western blotting. An unpaired t-test was used to assess statistical significance. **P < 0.01; ***P < 0.001; #, not significant.

To further evaluate the effects of SCP2 and cholesterol on PA cells in vivo, a PA xenograft model was generated by subcutaneous injection of either vector control or SCP2-overexpressing GH3 cells into nude mice. In addition, a diet-induced model of hypercholesterolemia was established through the feeding of a HCD beginning at the first injection [25]. The mice were randomly divided into five groups. After 9 days of injection, all mice were administered vehicle, ezetimibe or vismodegib daily for 3 weeks (Fig. 5a). Ezetimibe could reduce plasma cholesterol levels by inhibiting intestinal absorption of cholesterol [33]. Both SCP2 overexpression and HCD feeding promoted tumor growth, while ezetimibe and vismodegib inhibited tumor growth in vivo (Fig. 5b,c and d). Finally, the plasma cholesterol levels in the mice were measured, and we found the plasma cholesterol levels in the HCD-fed mice were significantly higher than those in mice with standard diet fed. Moreover, ezetimibe reversed the HCD-induced increase in plasma cholesterol levels (Fig. 5e). The western blotting results of excised tumors indicated that the upregulation of GLI1 protein level in SCP2 overexpression and HCD feeding groups, while ezetimibe and vismodegib could downregulate the GLI1 protein level in endogenous and exogenous modes, which indicating that both SCP2 overexpression and HCD feeding could activate Hh signaling pathway (Fig. 5f).

These results demonstrated that SCP2 promoted tumor growth by activating Hh signaling in vivo and hypercholesterolemia may be positively correlated with the tumor progression.

### Hypercholesterolemia was closely related to the occurrence of human PA

Since the results of the in vivo experiments suggested that hypercholesterolemia may be a risk factor for tumor progression, the correlation between plasma cholesterol levels and the occurrence of human PA was investigated. Plasma total cholesterol (TC), low-density lipoprotein cholesterol (LDL-C) and high-density lipoprotein cholesterol (HDL-C) levels in 100 PA patients and 140 healthy controls were analyzed. The mean age of the patients with PA and the healthy controls was 53.57 ± 9.95 and 49.99 ± 16.50, and the percentage of females was 40.00 and 43.57%, respectively. The incidence of hypercholesterolemia and increased LDL-C levels in PA patients were significantly higher than those in healthy controls, but the HDL-C levels did not differ (Table 1). These data suggested that hypercholesterolemia, especially an increased LDL-C level, might be related to the occurrence of PA.

**Table 1:** Comparing the occurrence of hypercholesterolemia in PA patients and healthy controls

	PA patients (*n* = 100)	Heathy controls (*n* = 140)	*P* value
Age (Mean ± SD)	53.57 ± 9.95	49.99 ± 16.50	0.055
Male/Female	60/40	79/61	0.581
TC increased (%)	22.00% (22/100)	12.14% (17/140)	0.041
LDL-C increased (%)	42.00% (42/100)	3.57% (5/140)	< 0.000
HDL-C increased (%)	2.00% (2/100)	2.86% (4/140)	0.675

*TC* total cholesterol, *LDL-C* low-density lipoprotein cholesterol, *HDL-C* high-density lipoprotein cholesterol

## Discussion

Little attention has been devoted to the potential role of cholesterol metabolism and key regulatory molecules in the progression of PA. We found that elevated SCP2 expression was correlated with the growth and proliferative activity of human PA samples and demonstrated that SCP2 overexpression promoted PA cell proliferation in vitro and in vivo by regulating abnormal membrane trafficking of cholesterol. Furthermore, we confirmed that cholesterol promoted tumor cell proliferation by directly activating the Hh signaling pathway and affecting the cell cycle and apoptosis. Moreover, we collected clinical information from PA patients and healthy controls and found that hypercholesterolemia might be related to the occurrence of PA. Our study first supported the correlation between cholesterol metabolism and PA, which led us to gain new insight into the mechanism of PA progression.

In our study, we initially found that SCP2 expression was higher in human PA samples than the normal pituitary gland and was positively correlated with tumor proliferative activity. Forced expression of SCP2 in PA cells promoted tumor growth, and inhibition of SCP2 suppressed the proliferation of PA cells. As a lipid transfer protein, SCP2 plays a key role in intracellular cholesterol movement by transporting cholesterol from intracellular sites, such as lipid droplets, to membranous organelles (mitochondria) and the plasma membrane [18]. Changes in levels or loss of SCP2 expression are associated with abnormalities in the intracellular trafficking and metabolism of cholesterol and other lipids [34, 35]. Recent evidence supports an oncogenic role of SCP2 in tumor. SCP2 has been reported to promote the proliferation of glioma cells by inhibiting apoptosis and inducing cell cycle progression through AKT-related signaling pathways [36]. In addition, the SCP2-specific inhibitor itraconazole slowed the trafficking of cholesterol from late endosomes and lysosomes to the plasma membrane by reducing the level of SCP2, resulting in repression of the AKT1-mTOR signaling pathway, induction of autophagy, and, ultimately, inhibition of cell proliferation in glioblastoma [19]. These results suggested that SCP2 promoted the proliferation of tumor cells, consistent with our findings. However, whether SCP2 affects tumor progression by regulating cholesterol metabolism remains unknown.

Subsequently, we found that SCP2 directly regulated intracellular cholesterol trafficking via the specific mechanism of transporting cholesterol from intracellular locations to the membrane without affecting the total cholesterol content of the cell. Additionally, a well-defined approach to increase the cholesterol level of the membrane in GH3 cells and primary human PA cells by treatment with the Mβ-CD/CHO complex was used to mimic the membrane cholesterol concentration. We found that increasing the membrane cholesterol content promoted PA cell proliferation. Changes in membrane cholesterol have been shown to affect tumor progression [11]. Lipid rafts, special small, cholesterol-rich lipid domains within the cell membrane, provide signal transduction platforms for oncogenic signaling pathways. Changes in cholesterol levels might lead to the structural modification of lipid rafts, resulting in activation or inhibition of raft-related proteins and affecting cell signaling [37], suggesting that membrane cholesterol might promote cell proliferation by affecting the activation of oncogenic signaling. Furthermore, we found that hypercholesterolemia significantly promoted the growth of tumors in a PA xenograft model experiment, while the cholesterol-lowering drug ezetimibe inhibited tumor growth. In addition, a statistical analysis of 100 PA patients showed that the incidence rate of hypercholesterolemia and the LDL-C level in PA patients were significantly higher than those in the 140 healthy controls. Hypercholesterolemia has long been considered an important clinical risk factor in several types of tumor [38]. The promotion of tumor growth and metastasis in a hypercholesterolemia model established by HCD feeding has been confirmed in vivo [39]. In addition, LDL-C has been reported to promote the proliferation and migration of breast cancer cells, suggesting that LDL-C may be a tumorigenic factor [40]. Ezetimibe was administered in an in vivo model of prostate cancer, showing antitumor effects through the inhibition of hypercholesterolemia-induced tumor angiogenesis [26]. These results suggest that cholesterol could promote the proliferation of PA cells and that hypercholesterolemia may be an important risk factor for PA.

Furthermore, we investigated the precise underlying mechanism by which abnormal cholesterol membrane trafficking induced the proliferation of PA cells. We found that cholesterol activated the Hh signaling pathway by downregulating PKA and SUFU expression, thus promoting GLI1 transcription, in GH3 cell lines and human primary PA cells. Recently, Huang et al. reported that exogenous cholesterol could directly activate the Hh signaling pathway by binding to the receptor SMO [29, 30]. The Hh signaling pathway has been thoroughly investigated in tumorigenesis and is related to many kinds of cancer, such as bladder, pancreatic, colorectal and prostate cancer [41]. Activation of Hh signaling, which can induce cell proliferation and hormone secretion, has been confirmed in pituitary stem cells [42]. Hh signaling has also been reported to be activated in human somatotropic adenoma samples [43]. In addition, activation of Hh signaling increases the activity of PA cells and the expression of target genes, while inhibition of GLI1 leads to the downregulation of target genes and induction of cell death [44]. PKA, SUFU and GLI1 are key molecules in the Hh signaling pathway. PKA can stabilize SUFU through phosphorylation to a form that is not subject to degradation induced by signaling activation [45]. SUFU plays a tumor-suppressive role by maintaining the inactivity of GLI transcription factors [46]. The GLI1 transcription factor can transduce activation signals into the nucleus and directly affect apoptosis and the cell cycle by regulating the expression of BCL2 and CCND1 [47]. Changes in apoptosis and the cell cycle are important reasons for the malignant proliferation of tumors [48, 49]. Consistent with this finding, decreasing the expression of the SMO receptor by shRNA, disrupting the function of the SMO receptor by vismodegib, and increasing the expression of PKA by forskolin inhibited cholesterol-induced cell proliferation and signaling activation. Similarly, the study on surgical human PA samples confirmed the activated Hh signaling which was consistent with previous publication [50]. However, which puzzled us that the PKA, a classical direct upstream activator of GLI1, was not detected significantly in human PA samples. It has been reported that SCP2 might activate AKT/mTOR pathway and Hh signaling could be inhibit by mTOR inactivation [36, 51, 52]. We further found that SCP2 could indirectly activate Hh signaling by increasing the phosphorylation of Akt/mTOR (Additional file 6: Figure S6A), while decreasing the phosphorylation of mTOR by rapamycin could inhibit the Hh signaling activation (Additional file 6: Figure S6B). Taken together, these results proposed that the activation of the Hh signaling induced by SCP2 is not unique, which provide us a direction for further study on the role of cholesterol metabolism in PA.

In summary, we first revealed that SCP2 expression was dysregulated in PA and demonstrated that SCP2-mediated abnormal cholesterol trafficking played an important role in PA growth. The membrane cholesterol concentration could promote the proliferation of PA by activating the Hh signaling pathway. In addition, hypercholesterolemia may be associated with the occurrence and progression of PA. Our results suggested that targeting cholesterol metabolism might be a potential treatment for patients with PA. However, the results of this study should be verified through further studies and clinical investigations.

## Conclusions

In conclusion, this study defined that cholesterol metabolism reprogramming also existed in PA and cholesterol could promote the proliferation of PA cells by activating Hh signaling, while inhibitors of signaling and cholesterol-lowering drug could suppress tumor growth. Together, these data offer new insight into the mechanism underlying PA progression and provide molecular mechanistic arguments for targeting cholesterol metabolism and Hh signaling in PA treatment.

## Supplementary information

**Additional file 1: Table S1.** Clinical characteristics of 40 human surgical PA samples.**Table S2.** Clinical characteristics and plasma cholesterol levels of 100 PA patients. **Table S3.** Clinical characteristics and plasma cholesterol levels of 140 healthy controls. **Table S4.** Patient clinical characteristics of primary human PA cells. **Table S5.** Primer list for qPCR.

**Additional file 2: Figure S2.** Inhibition efficiency assay and identification of primary human GH-producing PA cells. A. The inhibitory effect of different concentrations of itraconazole on SCP2 expression was assessed in GH3 cells by Western blotting. For subsequent experiments, 10 μM itraconazole was used, according to the expression levels of SCP2. B. The inhibitory effect of different concentrations of vismodegib on the Hh signaling pathway was assessed in GH3 cells by Western blotting. For subsequent experiments, 50 μM vismodegib was used, according to the expression levels of GLI1. C. SMO mRNA levels were measured in GH3 cells by RT-qPCR after transfection with shRNA. SMO-RNAi-2 and SMO-RNAi-3 were used for subsequent experiments, according to the expression levels of SMO (*n* = 3, ± SEM). D. Agonistic effects of different concentrations of forskolin on PKA expression were assessed in GH3 cells by Western blotting. For subsequent experiments, 25 μM forskolin was used, according to the expression levels of PKA. F. Cells derived from the primary GH-producing PA sample were identified by the presence of human GH. Green signal, GH staining; blue signal, DAPI nuclear staining. Scale bar, 50 μm. An unpaired t-test was used to assess statistical significance. *P < 0.05; #, not significant.

**Additional file 3: Figure S3.** Statistical analysis of the Western blotting results for different treatments. A. Statistical analysis of the Western blotting results in Fig. 3b (n = 3, ± SEM). B. Statistical analysis of the Western blotting results in Fig. 3c (n = 3, ± SEM). C. Statistical analysis of the Western blotting results in Fig. 3d (n = 3, ± SEM). D. Statistical analysis of the Western blotting results in Fig. 3e (n = 3, ± SEM). An unpaired t-test was used to assess statistical significance. *P < 0.05; **P < 0.01.

**Additional file 4: Figure S4.** Flow cytometry plots of cell apoptosis for different treatments. A. Flow cytometry plots of cell apoptosis in Fig. 2d. B. Flow cytometry plots of cell apoptosis in Fig. 3c. C. Flow cytometry plots of cell apoptosis in Fig. 3d. D. Flow cytometry plots of cell apoptosis in Fig. 3e.

**Additional file 5: Figure S5.** Flow cytometry plots of cell cycle for different treatments. A. Flow cytometry plots of cell cycle in Fig. 2e. B. Flow cytometry plots of cell cycle in Fig. 3c. C. Flow cytometry plots of cell cycle in Fig. 3d. D. Flow cytometry plots of cell cycle in Fig. 3e.

**Additional file 6: Figure S6.** SCP2 could indirectly activate the Hh signaling through AKT/mTOR pathway. A. Protein expression levels of p-AKT, AKT, p-mTOR, mTOR and GLI1 in the different groups (SCP2-OE, Vector) were assessed by Western blotting. B. GH3 cells were treated with different concentrations of rapamycin for 48 h. Protein expression levels of p-mTOR, mTOR and GLI1 were assessed by Western blotting.

**Additional file 7: Figure S7.** Negative control experiments of IHC in human PA samples. A. Incubation with PBS or isotype specific control IgG of rabbit. No SCP2-positive PA cells were observed. B. Incubation with PBS or isotype specific control IgG of rabbit. No PKA-positive PA cells were observed. C. Incubation with PBS or isotype specific control IgG of rabbit. No SUFU-positive PA cells were observed. D. Incubation with PBS or isotype specific control IgG of mouse. No GLI1-positive PA cells were observed. Scale bar, 50 μm.

## Data Availability

Not applicable.

## Abbreviations

AKT: Protein kinase B
BCL2: B cell lymphoma 2
cAMP: Cyclic adenosine monophosphate
CCK-8: Cell Counting Kit-8
CCND1: Cyclin D1
GH: Growth hormone
GLI1: Glioma-associated oncogene 1
HCD: High-cholesterol diet
HDL-C: High-density lipoprotein cholesterol
Hh: Hedgehog
HMGCR: HMG-CoA reductase
IHC: Immunohistochemical
LDL-C: Low-density lipoprotein cholesterol
mTOR: Mammalian Target of Rapamycin
Mβ-CD: Methyl-β-cyclodextrin
Mβ-CD/CHO: Methyl-β-cyclodextrin/cholesterol
PA: Pituitary adenomas
p-AKT: Phosphorylation-protein kinase B
PKA: Protein kinase A
p-mTOR: Phosphorylation-Mammalian Target of Rapamycin
SCP2: Sterol carrier protein 2
SMO: Smoothened
SUFU: Suppressor of fused
TC: Total cholesterol
