# Supplementary material for: SCP2-mediated cholesterol membrane trafficking promotes the growth of pituitary adenomas via Hedgehog signaling activation
Source: J Exp Clin Cancer Res. 2019 Sep 13;38:404. doi: 10.1186/s13046-019-1411-9 (PMC6743201; doi:10.1186/s13046-019-1411-9)
Supplement: Supplementary file 1 — Additional file 1: Table S1. Clinical characteristics of 40 human surgical PA samples.Table S2. Clinical characteristics and plasma cholesterol levels of 100 PA patients. Table S3. Clinical characteristics and plasma cholesterol levels of 140 healthy controls. Table S4. Patient clinical characteristics of primary human PA cells. Table S5. Primer list for qPCR. [file 13046_2019_1411_MOESM1_ESM.docx]

**Additional file 1: Table 1. Clinical characteristics of 40 human surgical PA samples.**

| Case | Age | Gender | Hormonal type | KI-67≥3% | P53 |
| --- | --- | --- | --- | --- | --- |
| 1 | 61 | M | NFPA | - | - |
| 2 | 20 | M | NFPA | - | - |
| 3 | 35 | M | NFPA | - | - |
| 4 | 63 | F | NFPA | - | - |
| 5 | 52 | M | NFPA | - | - |
| 6 | 60 | F | NFPA | - | - |
| 7 | 55 | F | NFPA | - | - |
| 8 | 64 | F | NFPA | - | - |
| 9 | 54 | F | PRL | - | - |
| 10 | 24 | F | PRL | - | - |
| 11 | 61 | F | PRL | - | - |
| 12 | 38 | F | PRL | - | - |
| 13 | 49 | M | GH | - | - |
| 14 | 50 | F | GH | - | - |
| 15 | 49 | F | GH | - | - |
| 16 | 61 | F | GH | - | - |
| 17 | 35 | F | GH | - | - |
| 18 | 30 | M | GH | - | - |
| 19 | 32 | F | GH | - | - |
| 20 | 48 | F | GH | - | - |
| 21 | 33 | M | NFPA | + | + |
| 22 | 44 | M | NFPA | + | + |
| 23 | 71 | M | NFPA | + | + |
| 24 | 25 | M | NFPA | + | + |
| 25 | 55 | M | NFPA | + | + |
| 26 | 45 | M | NFPA | + | + |
| 27 | 40 | M | NFPA | + | + |
| 28 | 33 | M | NFPA | + | + |
| 29 | 41 | M | NFPA | + | + |
| 30 | 68 | M | NFPA | + | + |
| 31 | 47 | M | NFPA | + | + |
| 32 | 48 | F | NFPA | + | + |
| 33 | 32 | F | NFPA | + | + |
| 34 | 53 | F | NFPA | + | + |
| 35 | 49 | F | NFPA | + | + |
| 36 | 32 | F | PRL | + | + |
| 37 | 29 | F | PRL | + | + |
| 38 | 28 | F | GH | + | + |
| 39 | 53 | M | GH | + | + |
| 40 | 45 | F | GH | + | + |

**Additional file 1: Table 2. Clinical characteristics and plasma cholesterol levels of 100 PA patients.**

| Case | Age | Gender | Hormonal type | TC Increased | LDL-C Increased | HDL-C Increased |
| --- | --- | --- | --- | --- | --- | --- |
| 1 | 64 | F | NFPA | - | - | - |
| 2 | 58 | M | NFPA | + | + | - |
| 3 | 63 | M | NFPA | - | - | - |
| 4 | 63 | M | NFPA | - | - | - |
| 5 | 37 | M | NFPA | - | - | - |
| 6 | 41 | F | NFPA | - | - | - |
| 7 | 54 | F | NFPA | + | + | - |
| 8 | 44 | F | NFPA | - | - | - |
| 9 | 34 | M | NFPA | - | - | - |
| 10 | 52 | F | NFPA | - | + | - |
| 11 | 55 | F | NFPA | + | + | - |
| 12 | 36 | F | NFPA | - | - | - |
| 13 | 67 | M | NFPA | - | - | - |
| 14 | 44 | M | NFPA | + | - | - |
| 15 | 38 | M | NFPA | - | + | - |
| 16 | 42 | F | NFPA | - | - | - |
| 17 | 33 | M | NFPA | - | + | - |
| 18 | 53 | F | NFPA | + | + | - |
| 19 | 56 | F | NFPA | + | + | - |
| 20 | 51 | M | NFPA | - | + | - |
| 21 | 47 | M | NFPA | - | + | - |
| 22 | 54 | M | NFPA | - | - | - |
| 23 | 42 | F | NFPA | - | + | - |
| 24 | 33 | F | NFPA | + | + | - |
| 25 | 53 | M | NFPA | - | - | - |
| 26 | 41 | M | NFPA | + | + | - |
| 27 | 37 | F | NFPA | - | - | - |
| 28 | 63 | M | NFPA | - | + | - |
| 29 | 53 | M | NFPA | - | - | - |
| 30 | 54 | M | NFPA | + | + | - |
| 31 | 57 | M | NFPA | - | + | - |
| 32 | 67 | M | NFPA | - | - | - |
| 33 | 44 | F | NFPA | - | - | - |
| 34 | 54 | M | NFPA | + | + | - |
| 35 | 65 | M | NFPA | - | - | - |
| 36 | 70 | M | NFPA | - | - | - |
| 37 | 54 | M | NFPA | - | + | - |
| 38 | 50 | M | NFPA | - | - | - |
| 39 | 76 | M | NFPA | - | + | - |
| 40 | 63 | M | NFPA | + | + | - |
| 41 | 47 | F | NFPA | - | - | - |
| 42 | 55 | M | NFPA | - | + | - |
| 43 | 52 | M | NFPA | - | - | - |
| 44 | 57 | M | NFPA | - | + | - |
| 45 | 40 | M | NFPA | - | + | - |
| 46 | 52 | M | NFPA | - | - | - |
| 47 | 55 | F | NFPA | - | - | - |
| 48 | 51 | F | NFPA | + | + | - |
| 49 | 63 | M | NFPA | - | - | - |
| 50 | 63 | M | NFPA | + | + | - |
| 51 | 49 | F | NFPA | - | - | - |
| 52 | 46 | F | NFPA | - | - | - |
| 53 | 57 | F | NFPA | + | + | - |
| 54 | 34 | F | NFPA | - | - | - |
| 55 | 43 | F | NFPA | + | + | - |
| 56 | 52 | M | NFPA | + | + | - |
| 57 | 48 | F | NFPA | - | - | - |
| 58 | 52 | M | NFPA | + | + | - |
| 59 | 65 | M | NFPA | - | - | - |
| 60 | 64 | M | NFPA | - | - | - |
| 61 | 54 | M | NFPA | - | + | - |
| 62 | 59 | M | NFPA | - | - | - |
| 63 | 64 | M | NFPA | - | - | - |
| 64 | 48 | M | NFPA | - | + | - |
| 65 | 48 | F | NFPA | - | - | - |
| 66 | 51 | F | NFPA | - | - | - |
| 67 | 48 | F | NFPA | - | - | - |
| 68 | 49 | F | NFPA | - | - | - |
| 69 | 42 | F | NFPA | + | + | - |
| 70 | 80 | M | NFPA | - | + | - |
| 71 | 62 | M | NFPA | - | - | - |
| 72 | 56 | M | NFPA | - | - | - |
| 73 | 43 | M | PRL | - | + | - |
| 74 | 32 | M | PRL | - | + | - |
| 75 | 65 | M | PRL | - | + | - |
| 76 | 30 | F | PRL | + | + | + |
| 77 | 50 | M | PRL | - | - | - |
| 78 | 53 | M | PRL | - | + | - |
| 79 | 50 | M | PRL | - | - | - |
| 80 | 37 | M | PRL | - | - | - |
| 81 | 46 | M | GH | - | - | - |
| 82 | 49 | M | GH | - | - | - |
| 83 | 50 | F | GH | - | - | - |
| 84 | 53 | M | GH | - | - | - |
| 85 | 45 | F | GH | - | - | - |
| 86 | 40 | F | GH | - | - | - |
| 87 | 64 | F | GH | - | - | - |
| 88 | 36 | F | GH | - | - | - |
| 89 | 53 | M | GH | - | - | - |
| 90 | 57 | M | GH | + | + | - |
| 91 | 49 | M | GH | + | + | - |
| 92 | 61 | F | GH | - | - | - |
| 93 | 59 | M | GH | - | - | + |
| 94 | 33 | F | GH | - | - | - |
| 95 | 69 | F | GH | + | - | - |
| 96 | 57 | F | GH | - | + | - |
| 97 | 46 | F | GH | - | + | - |
| 98 | 53 | M | GH | - | - | - |
| 99 | 37 | M | GH | - | - | - |
| 100 | 35 | F | GH | - | - | - |

**Additional file 1: Table 3. Clinical characteristics and plasma cholesterol levels of 140 healthy controls.**

| Case | Age | Gender | TC Increased | LDL-C Increased | HDL-C Increased |
| --- | --- | --- | --- | --- | --- |
| 1 | 61 | F | - | - | - |
| 2 | 30 | F | - | - | + |
| 3 | 67 | F | - | - | - |
| 4 | 45 | F | - | - | - |
| 5 | 51 | F | - | - | - |
| 6 | 36 | F | + | - | - |
| 7 | 54 | F | + | - | + |
| 8 | 47 | F | + | - | - |
| 9 | 60 | F | - | - | - |
| 10 | 48 | F | - | - | - |
| 11 | 26 | F | - | - | - |
| 12 | 26 | F | - | - | - |
| 13 | 26 | F | - | - | - |
| 14 | 29 | F | - | - | - |
| 15 | 50 | F | - | - | - |
| 16 | 67 | F | - | - | - |
| 17 | 83 | F | + | + | - |
| 18 | 52 | F | - | - | - |
| 19 | 34 | F | + | - | - |
| 20 | 72 | F | - | - | - |
| 21 | 72 | F | - | - | + |
| 22 | 34 | F | - | - | - |
| 23 | 56 | F | - | - | - |
| 24 | 63 | F | - | - | - |
| 25 | 63 | F | + | - | + |
| 26 | 41 | F | + | + | - |
| 27 | 78 | F | - | - | - |
| 28 | 75 | F | - | - | - |
| 29 | 40 | F | + | + | - |
| 30 | 42 | F | - | - | - |
| 31 | 79 | F | + | - | - |
| 32 | 66 | F | - | - | - |
| 33 | 36 | F | - | - | - |
| 34 | 37 | F | + | + | - |
| 35 | 56 | F | - | - | - |
| 36 | 63 | F | - | - | - |
| 37 | 49 | F | - | - | - |
| 38 | 61 | F | - | - | - |
| 39 | 50 | F | - | - | - |
| 40 | 61 | F | - | - | - |
| 41 | 49 | F | - | - | - |
| 42 | 62 | F | - | - | - |
| 43 | 81 | F | - | - | - |
| 44 | 31 | F | - | - | - |
| 45 | 23 | F | - | - | - |
| 46 | 22 | F | - | - | - |
| 47 | 62 | F | - | - | - |
| 48 | 41 | F | - | - | - |
| 49 | 50 | F | - | - | - |
| 50 | 69 | F | - | - | - |
| 51 | 51 | F | - | - | - |
| 52 | 41 | F | - | - | - |
| 53 | 74 | F | - | - | - |
| 54 | 53 | F | - | - | - |
| 55 | 28 | F | - | - | - |
| 56 | 55 | F | - | - | - |
| 57 | 86 | F | + | + | - |
| 58 | 31 | F | - | - | - |
| 59 | 31 | F | + | - | - |
| 60 | 48 | F | - | - | - |
| 61 | 34 | F | - | - | - |
| 62 | 36 | M | - | - | - |
| 63 | 51 | M | - | - | - |
| 64 | 39 | M | - | - | - |
| 65 | 34 | M | - | - | - |
| 66 | 47 | M | - | - | - |
| 67 | 59 | M | - | - | - |
| 68 | 62 | M | - | - | - |
| 69 | 40 | M | - | - | - |
| 70 | 41 | M | - | - | - |
| 71 | 46 | M | - | - | - |
| 72 | 32 | M | - | - | - |
| 73 | 74 | M | - | - | - |
| 74 | 68 | M | - | - | - |
| 75 | 53 | M | - | - | - |
| 76 | 34 | M | - | - | - |
| 77 | 29 | M | - | - | - |
| 78 | 54 | M | - | - | - |
| 79 | 38 | M | - | - | - |
| 80 | 28 | M | - | - | - |
| 81 | 55 | M | - | - | - |
| 82 | 81 | M | + | - | - |
| 83 | 47 | M | - | - | - |
| 84 | 51 | M | + | - | - |
| 85 | 67 | M | - | - | - |
| 86 | 43 | M | - | - | - |
| 87 | 59 | M | - | - | - |
| 88 | 43 | M | - | - | - |
| 89 | 36 | M | - | - | - |
| 90 | 53 | M | - | - | - |
| 91 | 38 | M | - | - | - |
| 92 | 61 | M | - | - | - |
| 93 | 20 | M | - | - | - |
| 94 | 19 | M | - | - | - |
| 95 | 50 | M | - | - | - |
| 96 | 20 | M | - | - | - |
| 97 | 51 | M | - | - | - |
| 98 | 88 | M | - | - | - |
| 99 | 31 | M | - | - | - |
| 100 | 66 | M | - | - | - |
| 101 | 61 | M | - | - | - |
| 102 | 56 | M | - | - | - |
| 103 | 50 | M | - | - | - |
| 104 | 44 | M | - | - | - |
| 105 | 44 | M | - | - | - |
| 106 | 19 | M | - | - | - |
| 107 | 43 | M | - | - | - |
| 108 | 30 | M | - | - | - |
| 109 | 76 | M | - | - | - |
| 110 | 44 | M | - | - | - |
| 111 | 51 | M | - | - | - |
| 112 | 70 | M | - | - | - |
| 113 | 45 | M | + | - | - |
| 114 | 40 | M | - | - | - |
| 115 | 25 | M | - | - | - |
| 116 | 65 | M | - | - | - |
| 117 | 35 | M | + | - | - |
| 118 | 75 | M | - | - | - |
| 119 | 57 | M | - | - | - |
| 120 | 69 | M | - | - | - |
| 121 | 58 | M | - | - | - |
| 122 | 61 | M | - | - | - |
| 123 | 45 | M | - | - | - |
| 124 | 70 | M | - | - | - |
| 125 | 50 | M | - | - | - |
| 126 | 45 | M | - | - | - |
| 127 | 65 | M | - | - | - |
| 128 | 63 | M | - | - | - |
| 129 | 17 | M | - | - | - |
| 130 | 26 | M | - | - | - |
| 131 | 38 | M | - | - | - |
| 132 | 69 | M | - | - | - |
| 133 | 34 | M | - | - | - |
| 134 | 37 | M | - | - | - |
| 135 | 58 | M | - | - | - |
| 136 | 78 | M | - | - | - |
| 137 | 37 | M | - | - | - |
| 138 | 63 | M | + | - | - |
| 139 | 63 | M | - | - | - |
| 140 | 70 | M | - | - | - |

**Additional file 1: Table 4. Patient clinical characteristics of primary human PA cells.**

| Case | Age | Gender | Hormonal type |
| --- | --- | --- | --- |
| 1 | 41 | M | GH |
| 2 | 35 | M | GH |
| 3 | 52 | F | GH |
| 4 | 22 | M | GH |
| 5 | 42 | F | GH |
| 6 | 54 | F | GH |
| 7 | 46 | M | GH |
| 8 | 58 | M | GH |

**Additional file 1: Table 5. Primer list for qPCR.**

| Gene name (Species) | Primer sequences (5'-3') |
| --- | --- |
| SCP2（human） | F:TCAGCAGTGGACCAGGCATGTGTTG |
|  | R:TGCTTCACTGGCCAAAATTGCTGCT |
| SCP2（rat） | F:CCTTCAGAACGCACCAGATT |
|  | R:TGATTGTGCAGTCAGCCTTC |
| SMO(rat) | F:GCAGTTCCTCGGCTGCCTC |
|  | R:AGCCTCCATTAGGTTAGTGCG |
| PKA（human） | F:AGCCCACTTGGATCAGTTTGA |
|  | R:GTTCCCGGTCTCCTTGTGT |
| SUFU(human) | F:CACGCCATCTACGGAGAGTG |
|  | R:GTACTTGACGATAGCGGTAACC |
| GLI1(human) | F:AGCGTGAGCCTGAATCTGTG |
|  | R:CAGCATGTACTGGGCTTTGAA |
| β-actin(human) | F:CATGTACGTTGCTATCCAGGC |
|  | R:CTCCTTAATGTCACGCACGAT |
| β-actin(rat) | F:GAGGGAAATCGTGCGTGAC |
|  | R:GCATCGGAACCGCTCATT |
