# Supplementary figures and images for: SCP2-mediated cholesterol membrane trafficking promotes the growth of pituitary adenomas via Hedgehog signaling activation
Source: J Exp Clin Cancer Res. 2019 Sep 13;38:404. doi: 10.1186/s13046-019-1411-9 (PMC6743201; doi:10.1186/s13046-019-1411-9)

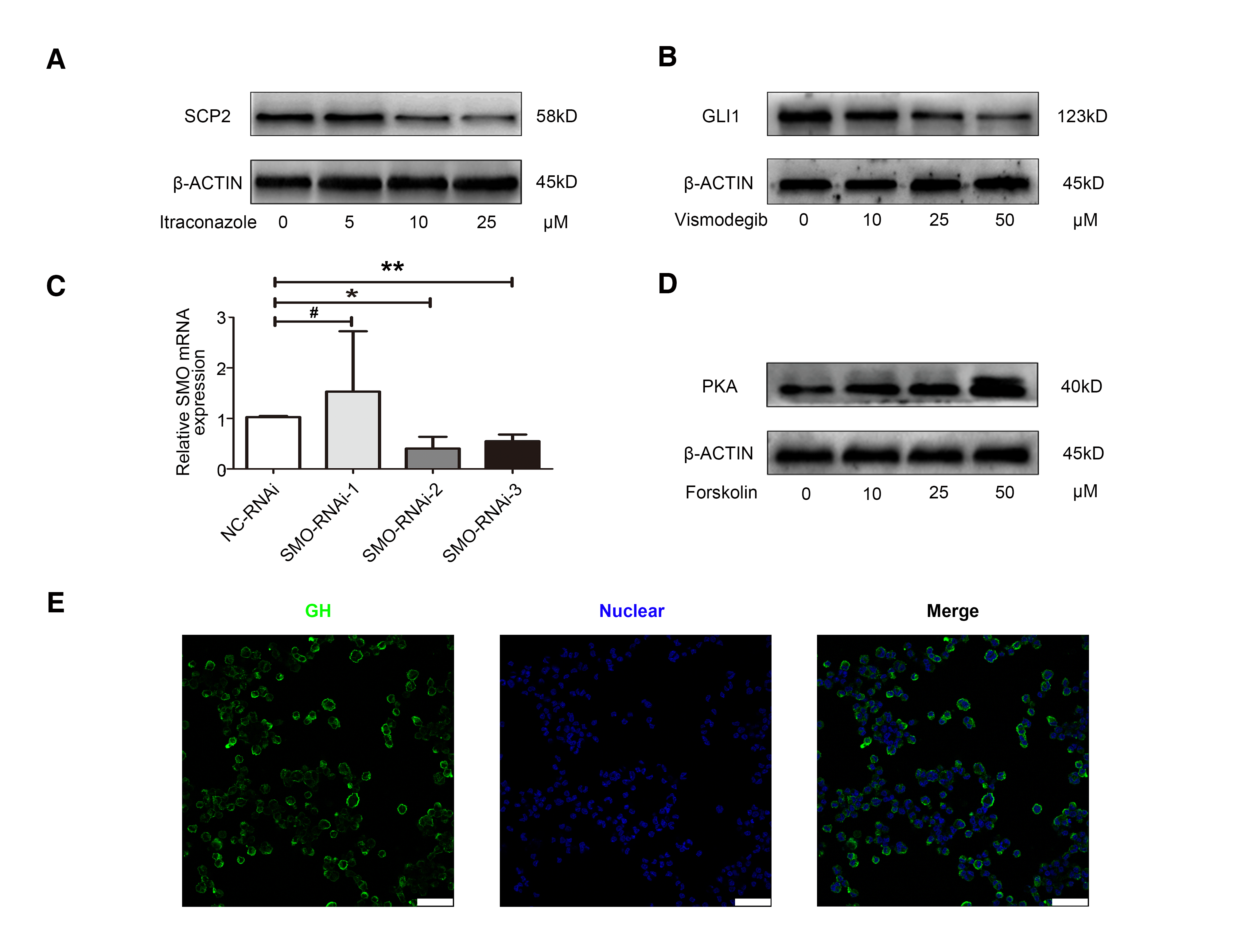

Supplement: Supplementary file 2 — Additional file 2: Figure S2. Inhibition efficiency assay and identification of primary human GH-producing PA cells. A. The inhibitory effect of different concentrations of itraconazole on SCP2 expression was assessed in GH3 cells by Western blotting. For subsequent experiments, 10 μM itraconazole was used, according to the expression levels of SCP2. B. The inhibitory effect of different concentrations of vismodegib on the Hh signaling pathway was assessed in GH3 cells by Western blotting. For subsequent experiments, 50 μM vismodegib was used, according to the expression levels of GLI1. C. SMO mRNA levels were measured in GH3 cells by RT-qPCR after transfection with shRNA. SMO-RNAi-2 and SMO-RNAi-3 were used for subsequent experiments, according to the expression levels of SMO (n = 3, ± SEM). D. Agonistic effects of different concentrations of forskolin on PKA expression were assessed in GH3 cells by Western blotting. For subsequent experiments, 25 μM forskolin was used, according to the expression levels of PKA. F. Cells derived from the primary GH-producing PA sample were identified by the presence of human GH. Green signal, GH staining; blue signal, DAPI nuclear staining. Scale bar, 50 μm. An unpaired t-test was used to assess statistical significance. *P < 0.05; #, not significant. [file 13046_2019_1411_MOESM2_ESM.tif]

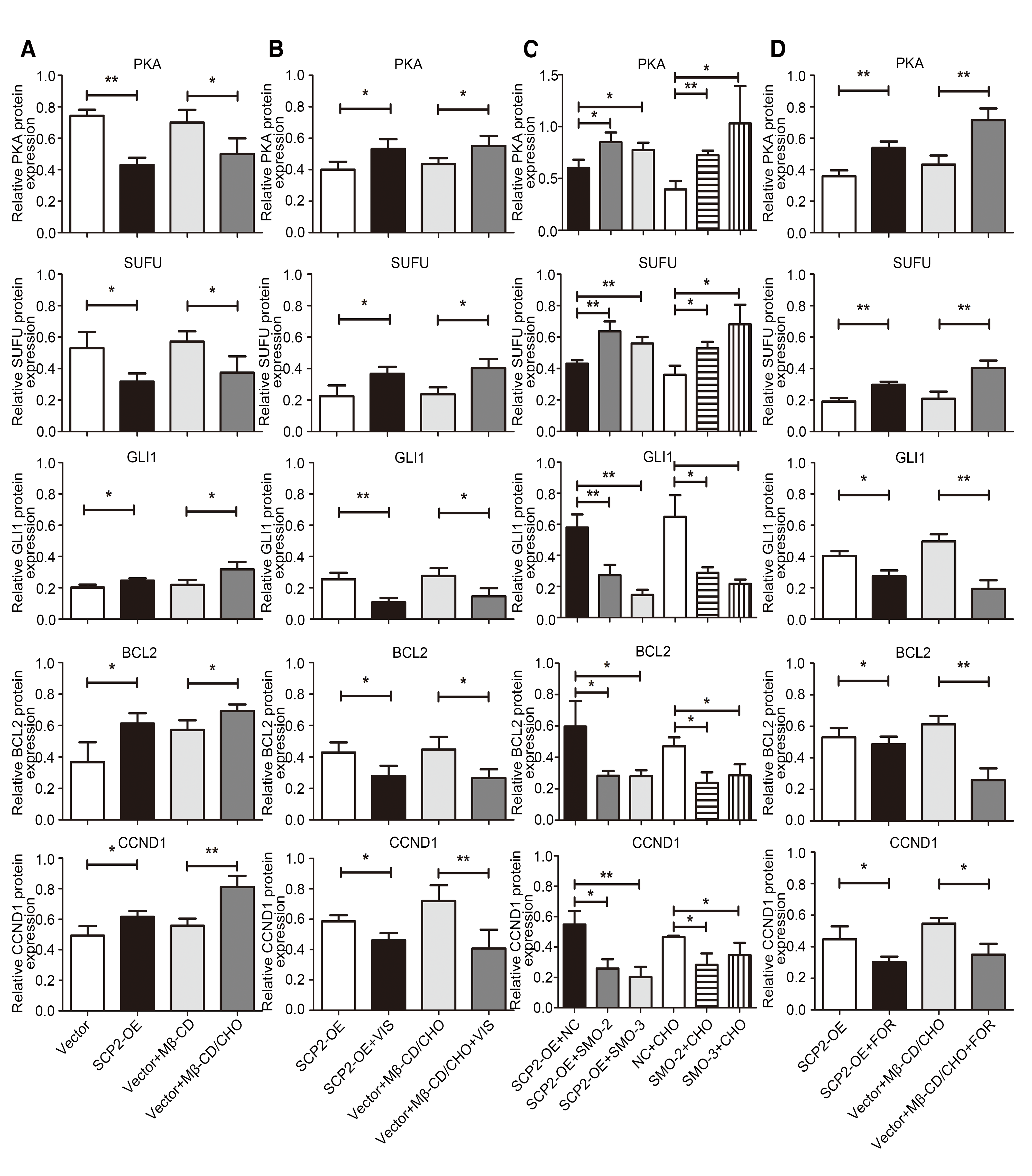

Supplement: Supplementary file 3 — Additional file 3: Figure S3. Statistical analysis of the Western blotting results for different treatments. A. Statistical analysis of the Western blotting results in Fig. 3b (n = 3, ± SEM). B. Statistical analysis of the Western blotting results in Fig. 3c (n = 3, ± SEM). C. Statistical analysis of the Western blotting results in Fig. 3d (n = 3, ± SEM). D. Statistical analysis of the Western blotting results in Fig. 3e (n = 3, ± SEM). An unpaired t-test was used to assess statistical significance. *P < 0.05; **P < 0.01. [file 13046_2019_1411_MOESM3_ESM.tif]

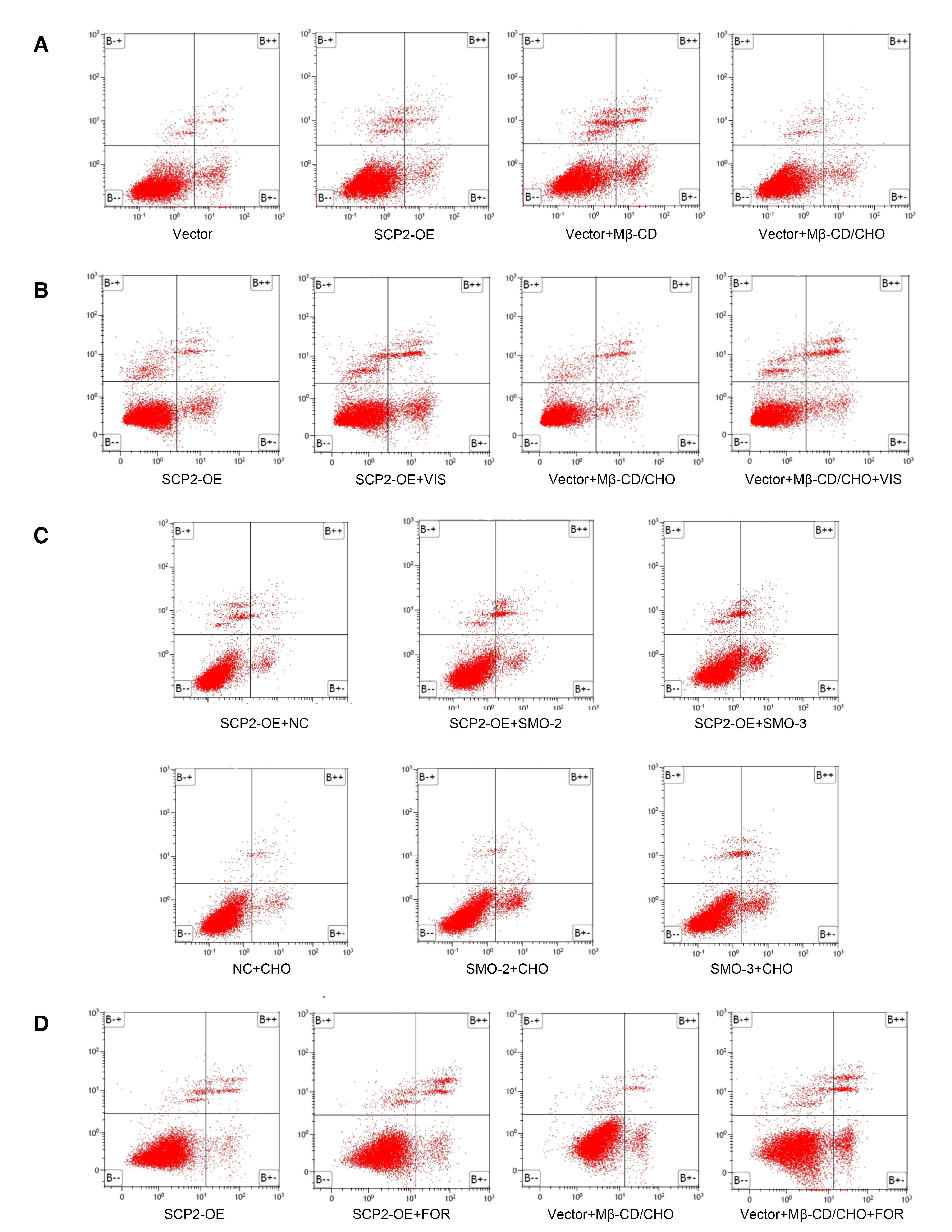

Supplement: Supplementary file 4 — Additional file 4: Figure S4. Flow cytometry plots of cell apoptosis for different treatments. A. Flow cytometry plots of cell apoptosis in Fig. 2d. B. Flow cytometry plots of cell apoptosis in Fig. 3c. C. Flow cytometry plots of cell apoptosis in Fig. 3d. D. Flow cytometry plots of cell apoptosis in Fig. 3e. [file 13046_2019_1411_MOESM4_ESM.tif]

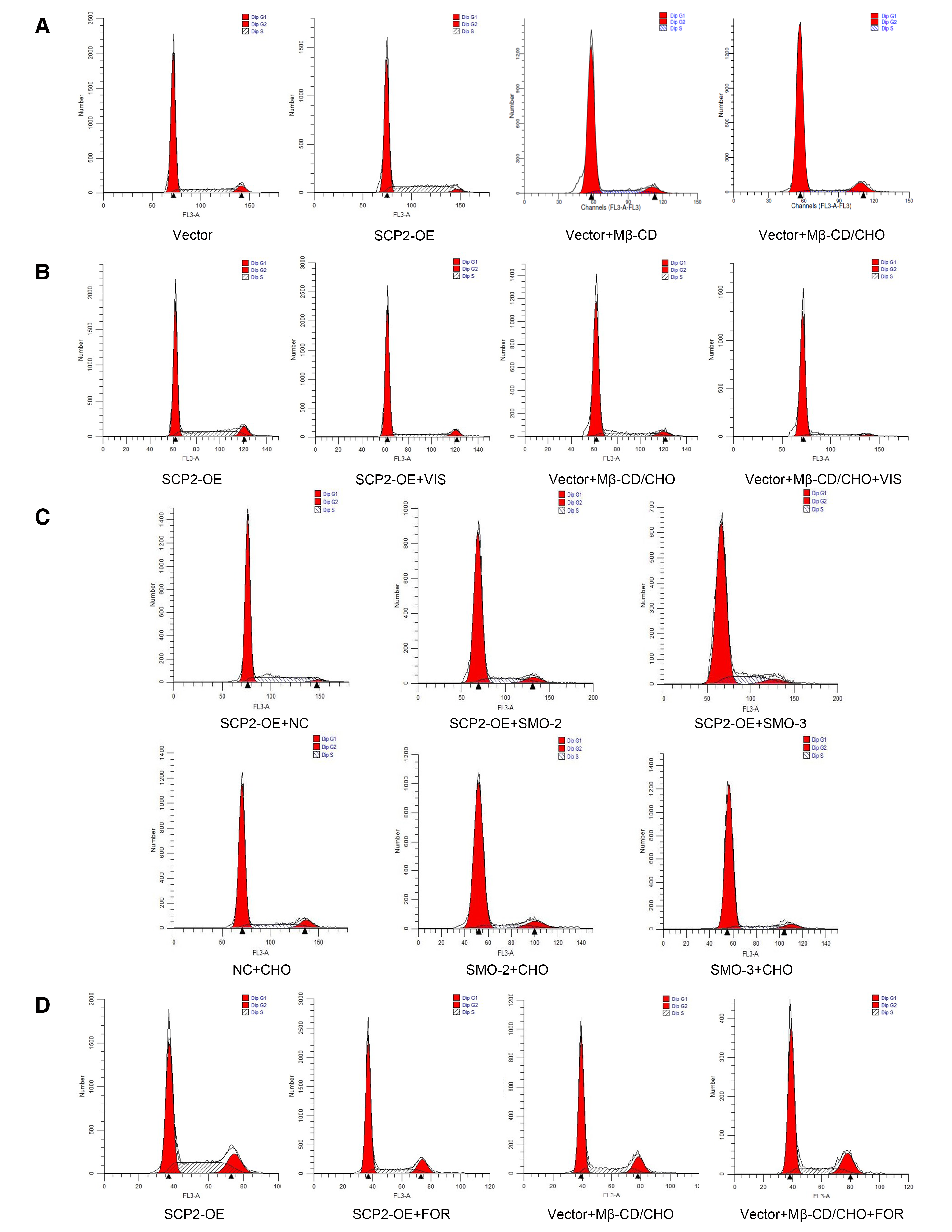

Supplement: Supplementary file 5 — Additional file 5: Figure S5. Flow cytometry plots of cell cycle for different treatments. A. Flow cytometry plots of cell cycle in Fig. 2e. B. Flow cytometry plots of cell cycle in Fig. 3c. C. Flow cytometry plots of cell cycle in Fig. 3d. D. Flow cytometry plots of cell cycle in Fig. 3e. [file 13046_2019_1411_MOESM5_ESM.tif]

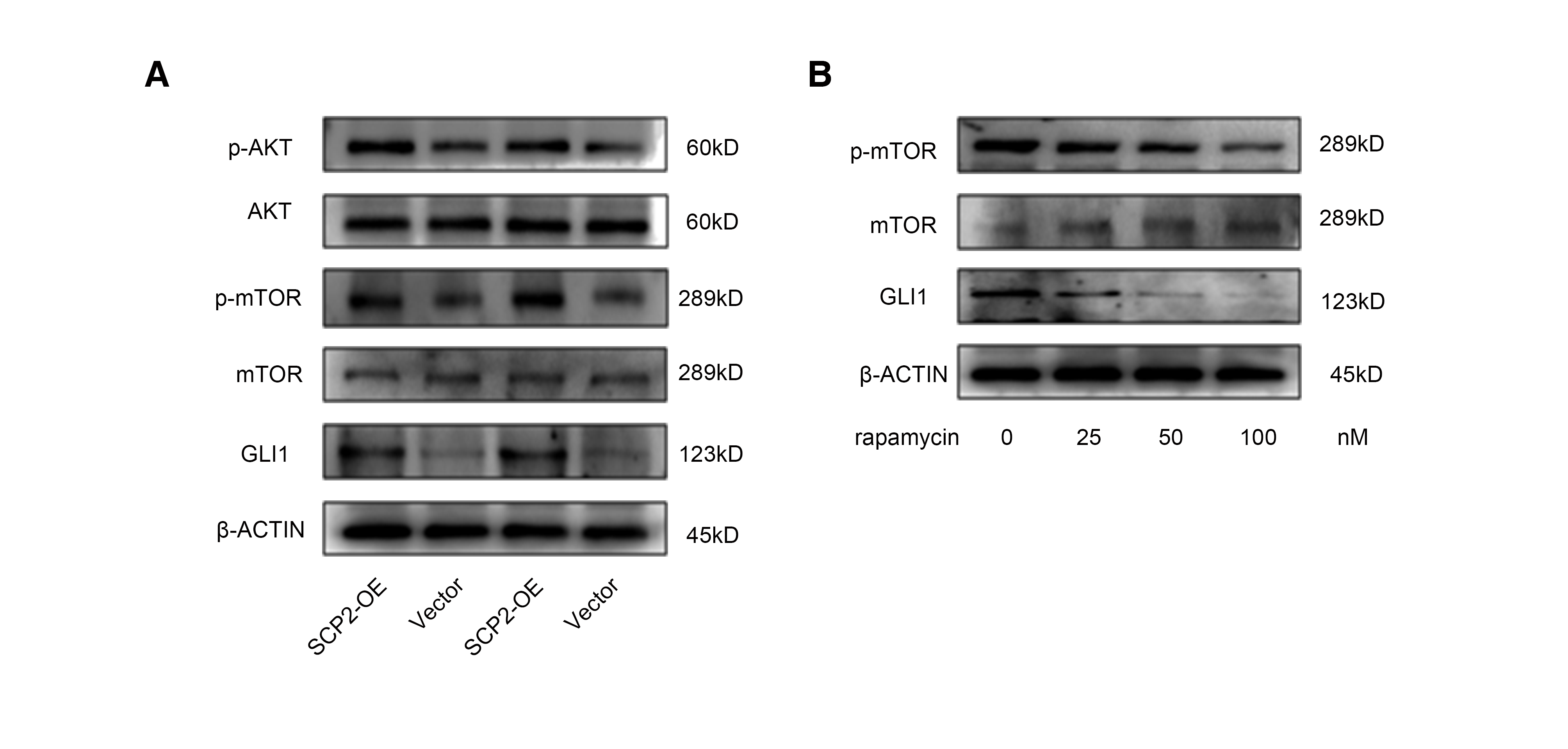

Supplement: Supplementary file 6 — Additional file 6: Figure S6. SCP2 could indirectly activate the Hh signaling through AKT/mTOR pathway. A. Protein expression levels of p-AKT, AKT, p-mTOR, mTOR and GLI1 in the different groups (SCP2-OE, Vector) were assessed by Western blotting. B. GH3 cells were treated with different concentrations of rapamycin for 48 h. Protein expression levels of p-mTOR, mTOR and GLI1 were assessed by Western blotting. [file 13046_2019_1411_MOESM6_ESM.tif]

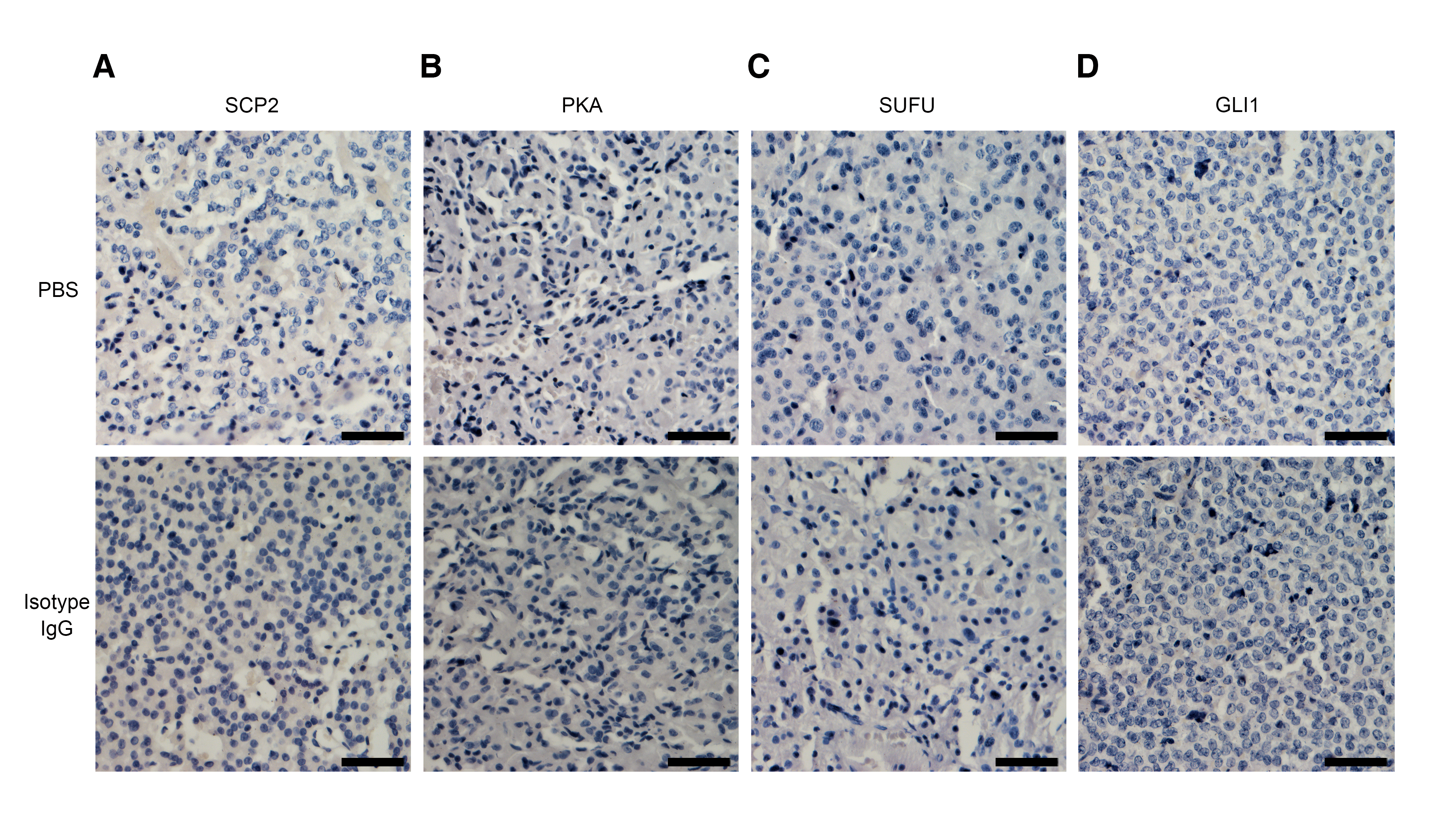

Supplement: Supplementary file 7 — Additional file 7: Figure S7. Negative control experiments of IHC in human PA samples. A. Incubation with PBS or isotype specific control IgG of rabbit. No SCP2-positive PA cells were observed. B. Incubation with PBS or isotype specific control IgG of rabbit. No PKA-positive PA cells were observed. C. Incubation with PBS or isotype specific control IgG of rabbit. No SUFU-positive PA cells were observed. D. Incubation with PBS or isotype specific control IgG of mouse. No GLI1-positive PA cells were observed. Scale bar, 50 μm. [file 13046_2019_1411_MOESM7_ESM.tif]
